# An annotated checklist of the amphibians and reptiles of North Padre Island, Texas, USA, with comparisons to adjacent barrier island and mainland herpetofauna

**DOI:** 10.3897/zookeys.1073.57241

**Published:** 2021-11-29

**Authors:** Mike Duran

**Affiliations:** 1 220 Rainbow Dr. №12083; Livingston, TX 77399, USA Unaffiliated Livingston United States of America

**Keywords:** Reptiles, amphibians, checklist, inventory, Padre Island, Texas, Mustang Island, North Padre Island, conservation, historical record, museum, iNaturalist

## Abstract

Padre Island is the world’s longest barrier island and includes the longest stretch of undeveloped barrier island in the world. Largely due to harsh environmental conditions and difficult access, only cursory and incomplete checklists and subjective estimates of abundance have been produced. The results of an inventory of amphibians and reptiles of North Padre Island conducted 2002–2020, including the results of extensive field surveys conducted 2002–2003, are reported herein. Natural history museum and iNaturalist records are summarized and compared among North and South Padre and Mustang islands and the mainland portion of the seven counties in which the islands occur. The conservation status of rare species and extirpation of others is noted. The morphology and taxonomic status of some unique occurrences are discussed. Eleven species of amphibians and 39 species of reptiles presently occur or have occurred naturally or as introduced or accidental species on North Padre Island. Twelve species of amphibians and 50 species of reptiles occur or have occurred on North Padre, South Padre, and Mustang islands. Thirty-one species of amphibians and 93 species of reptiles have been reported from the seven counties in which the islands occur.

## Introduction

**Figure 1.**
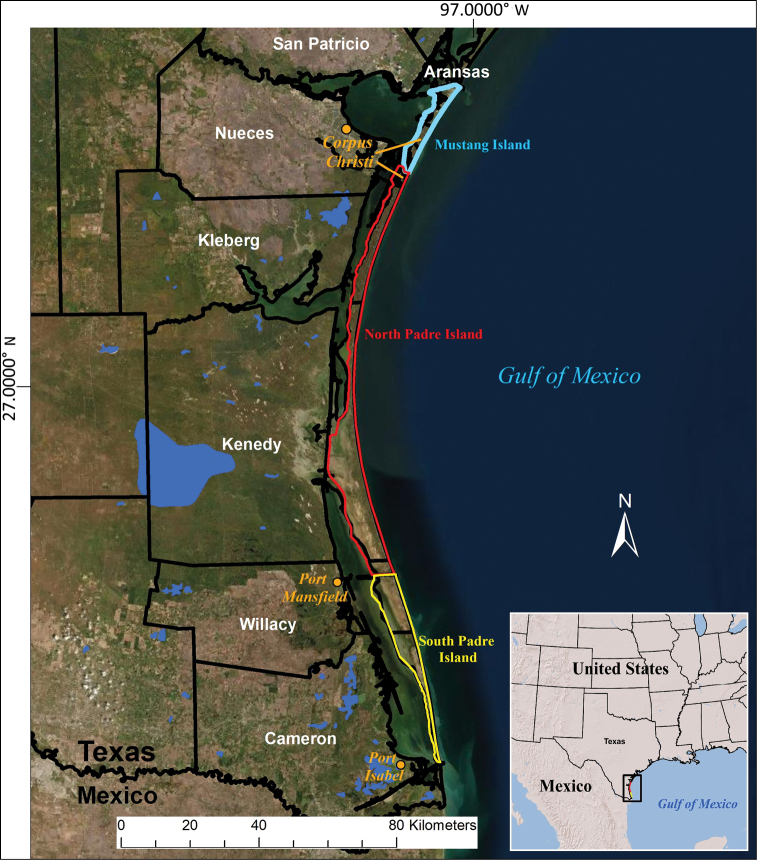
Map of seven-county study area including the South Texas barrier islands.

Extending for 178 km along the southern Texas coast, from Packery Channel in Corpus Christi to the Rio Grande River delta at the southern tip of the state, Padre Island is the world’s longest barrier island (Fig. 1; Tunnell and Judd 2002; National Park Service 2020). It is part of a barrier island chain that extends discontinuously along most of the Texas coast. Padre Island was divided into North and South Padre islands (NPI and SPI) when the Mansfield Channel was dredged in 1957. Historically, Mustang and Padre islands were separated by Corpus Christi Pass, before Packery Channel was dredged through the pass in 2005. North Padre Island is 122 km long and encompasses ~ 50,000 hectares. Including the frequently immersed tidal flats, the width of the island varies from ~ 275 m to ~ 11.5 km. The width of the consistently terrestrial part of the island varies from ~ 275 m to ~ 4.5 km. The northern 4.4 km of North Padre Island, within the Corpus Christi city limits, is urbanized and includes numerous saltwater canals and a few small man-made freshwater ponds. The southern 114 km of North Padre Island lies within the Padre Island National Seashore (PINS), established in 1962, which contains the longest undeveloped stretch of barrier island in the world (Tunnell and Judd 2002). Prior to this inventory, only cursory and incomplete checklists, based on limited or no field work, have been produced (Rabalais 1975; Baker and Rabalais 1978). This is the first inventory that includes extensive field work, as well as verification and enumeration of museum and other verifiable records.

### Formation of Padre Island and the Laguna Madre

Several models have been proposed to explain the formation of Padre and other barrier islands. The consensus among coastal geologists today, while recognizing that no one model exclusively explains barrier island formation, postulates that at the end of the Holocene, ~ 5,000–4,500 years ago, rising sea levels reached ~ 4.6 m above where they are today, and sandbars and shoals parallel to the shoreline began to form. When the sea reached its current level, ~ 2,800–2,500 years ago, those sandbars and shoals coalesced to form Padre Island and created bays and lagoons between the island and the mainland (LeBlanc and Hodgson 1959; Tunnel 2002).

Padre and other barrier islands are ever-changing as winds and currents regularly alter beaches, dunes, and tidal inlets. During extreme tidal events, freshwater wetlands and grasslands may be flooded with saline water from the Gulf of Mexico and hypersaline water from the inland lagoon. Dunes erode to depressions and flats. Storm surges sometimes alter the island suddenly, creating wash-over channels, closing or opening of passes, and flooding inland lagoons with sea water.

The lagoon formed inland of Padre Island, the Laguna Madre, was mostly isolated from the Gulf of Mexico prior to the dredging of Mansfield Channel in 1957 and received little freshwater inflow; together with the Laguna Madre de Tamaulipas, it became the largest of only six hypersaline bays and lagoons in the world (Javor 1989; Tunnel 2002). Prior to the dredging of the Gulf Intracoastal Waterway, the Laguna Madre was separated into the Upper Laguna Madre and Lower Laguna Madre by a frequently inundated land bridge, which appears on most maps as the Saltillo Flats, but is locally known by various names, including the Salt Flats, Kenedy Flats, Laguna Madre Flats, and most commonly, the Land Cut, a reference to the Intracoastal Waterway, which was cut through the flats in the 1940s. Salinity levels in the Upper Laguna Madre have been moderate in recent years: Olsen (2014) measured a mean salinity level of 44.1 psu (practical salinity units in parts per thousand concentration of sodium chloride) for the period of his study (1982–2012). Historically, the salinity of the upper Laguna Madre has varied from brackish (0.5–30 psu), after wet hurricanes, to brine (> 80 psu), during droughts, and has reached extremes over 100 psu, a level toxic to most organisms (Tunnel 2002). Due to relatively greater sea water and freshwater exchange, the salinity of the Lower Laguna Madre, while consistently hypersaline, has never risen to the briny extremes of the Upper Laguna Madre.

### Vegetation, ecological zones, and climate

Laine and Ramsey (1998) developed a Geographic Information System (GIS) coverage layer for PINS which grouped vegetation into 12 categories. They calculated that ~ 28% of landscape within PINS was composed of areas that were mostly not vegetated by vascular plants (thus mostly unsuitable for amphibians and reptiles), including algal flats (~ 22%), unconsolidated shore (~ 5%), and wash-over channels (~ 1%). They calculated that 22% of PINS occurred within the Laguna Madre. Of the remaining habitats, Laine and Ramsey (1998) calculated that 45.6% are classified as emergent wetlands and inland water, 28.5% are classified as grasslands, and 12.5% are classified as sparsely vegetated. Nelson et al. (2000) reported 456 species of plants in 77 families and 259 genera from PINS. They reported that the five most common families are Poaceae (20.8%), Asteraceae (12.5%), Fabaceae (9.6%), Cyperaceae (6.4%), and Euphorbiaceae (4.2%). Diamond et al. (2017) added 36 new species to the PINS plant list and delineated 16 terrestrial plant associations within PINS, among which grasslands made up 48.0% of the vegetation, tidal saline vegetation made up 33.19%, dune and foredune communities together made up 9.37%, and herbaceous wetlands made up 8.74%. A few shrublands and woodlands accounted for < 1% of the vegetation.

**Figure 2.**
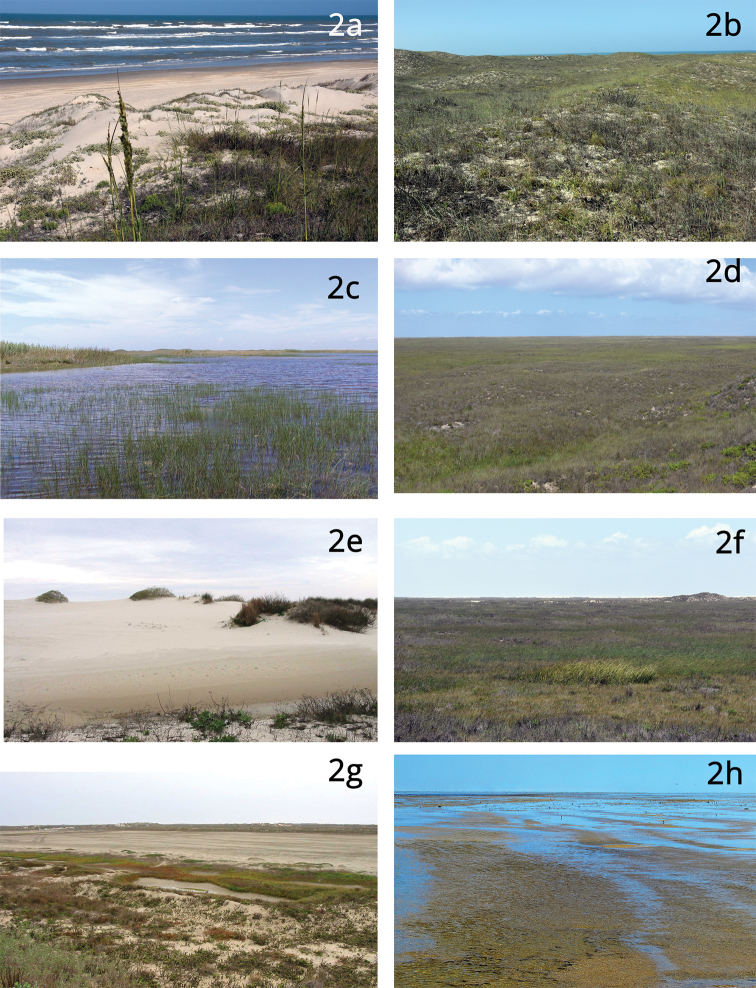
Major ecological zones of North Padre Island **a** back beach and foredunes **b** dune/swale complex **c** emergent wetlands **d** mid-island grasslands **e** sand dunes (back island dunes) **f** grassland/wetland matrix **g** saline flats **h** wind tidal flats (photograph by John Karges).

While plant communities on North Padre Island are generally interdigitating and unevenly distributed, an idealized spatial vegetation profile is helpful to visualize the distribution of ecological zones from the Gulf of Mexico, proceeding westward to the Laguna Madre (adapted from Judd et al. 1977 and Diamond et al. 2017):

1. A forebeach zone at the shoreline of the Gulf of Mexico is unvegetated but sometimes partially to entirely littered with planktonic marine plants in the genus Sargassum.
2. A Gulf facing, sparsely vegetated, back-beach and foredune zone (Fig. 2a), usually not more than 50 m wide, within which the most conspicuous vegetation includes beach morning-glory (Ipomoea imperati), shoreline purslane (Sesuvium portulacastrum), and goat’s foot convolvulus (I. pes-caprae). Grasses and forbs such as sea oats (Uniola paniculata) and beach evening primrose (Oenothera drummondii) are also apparent among patches of bare sand.
3. Just westward of the foredunes, a moderately vegetated rolling dune/swale complex (Fig. 2b) occurs where dunes that may reach 10 m elevation or more surround interstitial wetter swales. This complex includes at least three plant associations that make up ~ 8.2% of island vegetation (Diamond et al. 2017). Among the most conspicuous plants in this zone are sea oats (U. paniculata), camphorweed (Heterotheca subaxillaris), wooly croton (Croton punctatus), and partridge pea (Chamaecrista fasciculata.
4. An emergent wetland zone (Fig. 2c), most apparent on the northern part of the island, occurs as low-lying mid-island flats and depressions or ephemeral pools and lakes. Semi-aquatic and salt-tolerant plants apparent in this zone include cattails (Ty pha domingensis), cordgrass (Spartina sp.), and sedges, including common threesquare (Schoenoplectus pungens), beaksedges (Rhynchospora sp.), umbrella-sedges (Fuirena sp.), and fimbrys (Fimbristylis sp.). This zone includes ~ 16 ha of near-monoculture stands of the invasive common reed (Phragmites australis), which mostly occurs in and around a narrow 6-km ephemeral lake that begins ~ 22 km south of Packery Channel, but it is present in many wet areas throughout the island.
5. Mid-island grasslands (Fig. 2d), where seacoast bluestem (Schizachyrium littorale), gulfdune paspalum (Paspalum monostachyum), and bushy bluestem (Andropogon glomeratus) are dominant. The Seacoast Bluestem – Gulfdune Paspalum Grassland plant association makes up ~ 48% of island vegetation (Diamond et al. 2017). The generalized profile does not adequately describe the spatial setting of grasslands, which often occur as an overlapping matrix with emergent wetlands (Fig. 2e). Padre Island grasslands are infrequently flooded and relatively species-rich mid-island rolling flats dotted with low (mostly 0.5–1.5 m) vegetated dunes.
6. Sand dunes (Fig. 2f), sometimes called “back-island dunes,” mostly occur within a few hundred meters of the Laguna Madre but may occur in any part of the island. They are composed of deep, dry, and shifting sands and are sparsely vegetated to unvegetated.
7. Saline flats (Fig. 2g) are irregularly tidally inundated and range from sparsely vegetated to relatively densely vegetated with halophytic species such as bushy sea oxeye (Borrichia frutescens), turtleweed (Batis maritima), saltgrass (Distichlis spicata), and shoregrass (Monanthochloe littoralis.
8. Wind tidal flats (Fig. 2h) are unvegetated or covered with a mat of the alga Lyngbya confervoides.
9. The Laguna Madre, a hypersaline lagoon, the ecological health of which depends on the health of seagrass meadows. According to Onuf (2006), ~ 75% of the substrate of the Laguna Madre is covered by seagrasses, mostly shoal grass (Halodule wrightii) and manatee grass (Syringodium filiforme).

According to NOAA (2021), Padre Island becomes considerably drier from the northern end at Corpus Christi, which receives 80.5 cm of rainfall per year, to the mid-point at Port Mansfield which receives 65.8 cm per year. Port Isabel, at the southern end of Padre Island, receives ~ 73.4 cm per year. The vegetation of North Padre Island gradually becomes less dense, north to south, i.e., the northern end vegetation is relatively lush and dense, while much of the landscape near either side of the Mansfield Channel is sparsely vegetated with extensive areas of bare sand.

Average annual temperatures become slightly warmer, north to south, with Corpus Christi at 22.3 °C, Port Mansfield 22.8 °C, and Port Isabel 23.1 °C. In some years, temperatures never drop below freezing. Most of the rainfall occurs in late summer and early fall, particularly at the southern end, where Port Isabel receives ~ 35% of its annual rainfall in September and October (NOAA 2021; https://www.ncdc.noaa.gov/cdo-web).

## Materials and methods

A review of the relevant literature and museum specimens was conducted, through 08 October 2020, for the five counties in which the islands occur (Cameron, Kenedy, Kleberg, Nueces, Willacy) and for the two counties adjacent to Corpus Christi Bay (Aransas and San Patricio; hereafter “the seven counties”). Museum records were reviewed in September 2021, and new species added after 08 October 2020 are included. Research grade iNaturalist records for the South Texas barrier islands were compiled through 08 October 2020. Research grade iNaturalist observations of new species added between 08 October 2020 and 12 September 2021 are also included. For the most recent review of the museum database, I obtained most of the records through Vertnet.org (2021). The museum and iNaturalist searches of the seven counties captured a few records from San José Island, Harbor Island, South Bird Island, and other islands in the Laguna Madre and Corpus Christi Bay. Those records are labelled “other islands” (**OI**).

For the 2002–2003 surveys, I used ArcView 3.3 with the Hawth Tools extension, Random, to randomly select fifteen study sites on North Padre Island within six ecological zones (see previous section for discussion of ecological zones) within three geographical zones. Herpetofauna were sampled in six ecological zones: 1) back-beach and foredunes, 2) dune/swale complex 3) grasslands, 4) wetlands, 5) grassland/wetland complex, and 6) sand dunes. Herpetofauna were not sampled on the forebeach, saline flats, or wind tidal flats. The northernmost study site, just inside the PINS boundary, 13.8 km south of Packery Channel, was 102 km north of the southernmost study site, 250 m north of the Mansfield Channel. Two sites were selected non-randomly so that wetlands would be adequately sampled. The original design was based on ecological zones delineated by Laine and Ramsey III (1998), the only spatial vegetation layer available at that time. Subsequent vegetational analyses, all references to plant associations, and scientific and common names for plants of PINS, follow Diamond et al. (2017). Unless another source is cited, distance and area measurements were calculated using ArcGIS 10.8 (ESRI 2011).

Aquatic surveys were conducted opportunistically at all permanent ponds and most ephemeral pools from ~ 18 km south of Packery Channel to ~ 27 km south of Packery Channel and at ephemeral pools that occurred within or near the randomly selected study sites. During 2002 and 2003, road-searching and calling frog surveys were conducted from the southern end of Park Road 22, north to the intersection of Park Road 22 with State Highway 360. Calling-frog surveys were conducted and audio-recorded opportunistically (during and after heavy rainfall), and at predetermined points for 10 minutes (after the methodology of Mossman et al. 1998; North American amphibian monitoring program protocol 2012). Seines, dip nets, and funnel-type minnow traps were used to sample tadpoles, sirens, and newts. Turtles were trapped in hoop traps baited with sardines and chicken livers and were observed with binoculars and verified with photos. Calling-frog, road searching, and some visual encounter surveys continued, intermittently, into 2019.

**Figure 3.**
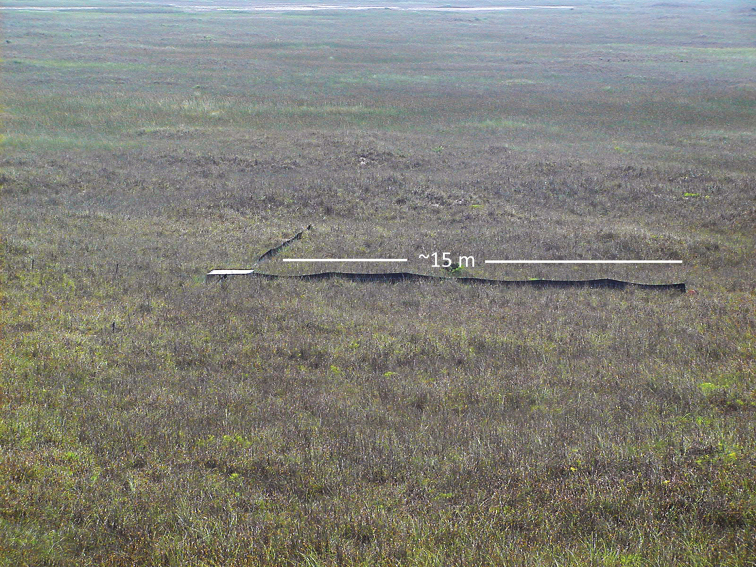
Reptile and amphibian trap array in grassland near the midpoint of North Padre Island. Two of four ~ 15 m arms of the array have been installed. *Cemophoralineri* (Texas scarletsnake) was captured in the center box at this study site.

Field work and trap installation and removal was performed by a team that consisted of The Nature Conservancy and National Park Service (**NPS**) personnel and volunteers. For terrestrial and semi-aquatic species, our team installed two variations of drift-fence/pitfall arrays. The first consisted of three equally spaced drift-fence arms, which originated at a center 19-liter pitfall buried to the rim and extended 7 m to 19-liter pitfalls at the ends and at midpoints, so that each array included seven pitfalls. The second type of array consisted of a 1.2 m × 1.2 m × 45.7 cm box, constructed after the design of Burgdorf et al. (2005), with a plywood top and bottom, hardware cloth wrapped around the sides, and funnel entrances built from hardware cloth on each of the four sides of the box (Fig. 3). A drift fence originating at each funnel opening extended approximately 15 m from the box. A 19-liter pitfall was buried to the rim at the end of each fence. Drift fences, fashioned from hardware cloth or nylon erosion-control fencing, were 91.4 cm high and were buried 20–25 cm in the ground. Traps were opened periodically May-October 2002. Turtle trapping was conducted in May 2003. Visual encounter surveys were conducted along transects within a square kilometer surrounding the study site random point. Visual encounter surveys were also conducted opportunistically and non-randomly throughout the island in areas which may have been under-sampled by the random-sampling protocol. Voucher specimens for each species and any specimens found dead on roads or in traps were collected and deposited in the University of Texas Biodiversity Collections (**TNHC**) or the Biodiversity Research and Teaching Collections at Texas A&M University (**TCWC**). Only tissue was collected from federally or state listed species. Most captured animals were photographed.

Scientific and common names follow Crother (2017), except for the genera *Bufo* (Pauly et al. 2009), *Masticophis* (Myers et al. 2017), and *Rana* (Yuan et al. 2016).

This work was performed under the authority of Texas Parks and Wildlife Department scientific research permit SPR-0302-204, US National Park Service scientific research and collecting permits PAIS-2003-SCI-0008, PAIS-2009-SCI-0012, and PAIS-2014-SCI-0006, and US Fish and Wildlife Service permit TE820085-0.

Abbreviations used in this paper are as follows:

**AMNH** American Museum of Natural History

**ASNHC** Angelo State Natural History Collection

**BPP** Bayesian Phylogenetics and Phylogeography

**GIS** Geographic information system

**NOAA** National Oceanic and Atmospheric Administration

**NPI** North Padre Island

**NPS** US National Park Service

**OI** other islands

**PINS** Padre Island National Seashore

**SPI** South Padre Island

**TAMUK** Texas A&M University at Kingsville

**TCWC** Biodiversity Research and Teaching Collections at Texas A&M University

**TNHC** University of Texas Biodiversity Collections

**UMMZ** University of Michigan Museum of Zoology

**URL** Uniform Resource Locator (link)

**VSR** Ventral scale rows

## Results

Forty-four institutions held 14,830 specimens from the seven counties including 1751 specimens from the South Texas barrier islands held by 26 institutions and 47 specimens held by six institutions from San José Island, Harbor Island, South Bird Island, and other small man-made and natural islands in the Laguna Madre and Corpus Christi Bay (Table 1, 2; Suppl. material 1). There were 4904 iNaturalist research grade observations from the South Texas barrier islands as of 08 October 2020.

**Table 1.** Museums which contain amphibian and reptile specimens from the mainland portion of the seven adjacent counties (**ML**), Mustang Island (**MI**), North and South Padre (**NPI** and **SPI**), other small islands in the Laguna Madre and Corpus Christi Bay adjacent to Mustang Island (**OI**).

Inst code	Institution name	ML	MI	NPI	OI	SPI	Total
AMNH	American Museum of Natural History	4 900	32	338	28	51	**5 349**
TNHC	U. of Texas at Austin - Texas Natural History Collections	1 882	232	55	13	13	**2 195**
TCWC	Texas A&M U. Biodiversity Research and Teaching Collections	1 306	76	151	3	18	**1 554**
USNM	National Museum of Natural History, Smithsonian Institution	616	8	46	1	6	**677**
BUMMC	Baylor University, Mayborn Museum Complex	472	7	17		50	**546**
UTA	U. of Texas at Austin - Texas Natural History Collections	371	18	12		63	**464**
ASNHC	Angelo State Natural History Collection	108	19	315			**442**
KU	University of Kansas Biodiversity Institute	389	22	1	1	8	**421**
LSUMZ	Louisiana State University Museum of Natural Science	396	4	5		1	**406**
UMMZ	University of Michigan Museum of Zoology	404		1		1	**406**
UF	Florida Museum of Natural History	311	6	1	1		**319**
MVZ	Museum of Vertebrate Zoology, UC Berkeley	113	169	3			**285**
FMNH	Field Museum of Natural History	277					**277**
MCZ	Museum of Comparative Zoology, Harvard University	204		7			**211**
CM	Carnegie Museum of Natural History	156	5	11			**172**
SDNHM	San Diego Natural History Museum	162	2	1			**165**
LACM	Natural History Museum of Los Angeles County	100	16	1			**117**
UCM	University of Colorado Museum of Natural History	83	12	2			**97**
UMNH	Natural History Museum of Utah	72				1	**73**
ANSP	Academy of Natural Science Philidelphia	65					**65**
CHAS	Chicago Academy of Sciences	65					**65**
UBCBBM	University of British Columbia Beaty Biodiversity Museum	59					**59**
NCSM	North Carolina Museum of Natural Sciences	57					**57**
CAS	California Academy of Sciences	54	1				**55**
CUMV	Cornell University Museum of Vertebrates	49				1	**50**
MSUM	Michigan State University Museum	45					**45**
UAZ	University of Arizona Museum of Natural History	44					**44**
UTEP	University of Texas at El Paso Biodiversity Collections	36	1	1			**38**
BYU	Monte L. Bean Museum, Brigham Young University	29				2	**31**
OMNH	Sam Noble Oklahoma Museum of Natural History	17				7	**24**
CLO	Macaulay Library Audio and Video Collection	21					**21**
AUM	Auburn University Museum of Natural History	19					**19**
FHSM	Fort Hays Sternberg Museum of Natural History	10	6	1			**17**
SLU	Southeastern Louisiana University	8	2				**10**
GSU	Georgia Southern University	7	1				**8**
NBMB	New Brunswick Museum	8					**8**
ISM	Illinois State Museum	7					**7**
MSB	Museum of Southwestern Biology	7					**7**
PMNS	Perot Museum of Nature and Science	5		2			**7**
BSNS	Buffalo Society of Natural Sciences	5					**5**
MPM	Milwaukee Public Museum	5					**5**
YPM	Yale Peabody Museum	3		0			**3**
PBDB	Paleobiology Database	2					**2**
OSUM	Sam Noble Oklahoma Museum of Natural History	2					**2**
**Total**		**12 951**	**639**	**971**	**47**	**222**	**14 830**

**Table 2.** Museum and iNaturalist records of amphibians and reptiles of the South Texas barrier islands. **ML** = mainland; **MI** = Mustang Island; **NPI** = North Padre Island; **SPI** = South Padre Island; **OI** = San José Island (in Aransas Co.) and other natural and manmade islands in the Laguna Madre and Corpus Christi Bay.

Taxon	iNaturalist Observations					Museum specimen records					All totals
	MI	NPI	SPI	iNatTot	MI	ML	NPI	OI	SPI	MusTot	
AMPHIBIA	144	206	37	387	144	5 789	64	3	33	6 033	6 420
Anura	144	206	37	387	144	4 797	64	3	33	5 041	5 428
Bufonidae		30	18	94	132	1 028	9			1 169	1 263
*Bufocognatus*						4				4	4
*Bufodebilis*						38				38	38
*Bufohorribilis*						2				2	2
*Bufonebulifer*	46	18	18	82	8	447	7			462	544
*Bufospeciosus*					124	528				652	652
*Bufowoodhousii*		12		12		8	2			10	22
*Rhinellamarina*						1				1	1
Eleutherodactylidae	3		4	7		184			3	187	194
*Eleutherodactylusplanirostris*			2	2					2	2	4
*Eleutherodactyluscampi*	3		2	5		184			1	185	190
Hylidae	64	95	14	173	3	1 052	15		28	1 098	1 271
*Acrisblanchardi*						72				72	72
*Hylacinerea*	9	21		30	1	486	14			501	531
*Hylasquirella*	55	66	10	131	2	80			28	110	241
*Hylaversicolorcomplex*						19				19	19
*Osteopilusseptentrionalis*			1	1							1
*Pseudacrisclarkii*		8		8		239	1			240	248
*Pseudacrisstreckeri*						80				80	80
*Smiliscabaudinii*				3		76				76	79
Microhylidae	14	11		25		512	2		2	516	541
*Gastrophrynecarolinensis*	14	9		23		3	2			5	28
*Gastrophryneolivacea*		2		2		364				364	366
*Hypopachusvariolosus*						145			2	147	147
Ranidae	16	9	1	26	4	851	8	2		865	891
*Ranaberlandieri*	14	7	1	22	3	597	7			607	629
*Ranacatesbeianus*	2	1		3	1	76				77	80
*Ranasphenocephala*		1		1		178	1	2		179	180
*Scaphiopodidae*	1	61		62	5	1 170	30	1		1 206	1 268
*Scaphiopuscouchii*						794				794	794
*Scaphiopushurterii*	1	61		62	5	235	30	1		271	333
*Speabombifrons*						90				90	90
*Speamultiplicata*						51				51	51
Caudata						992				992	992
Ambystomatidae						132				132	132
*Ambystomamavortium*						132				132	132
Salamandridae						217				217	217
*Notophthalmusmeridionalis*						216				216	216
*Notophthalmusviridescens*						1				1	1
Sirenidae						643				643	643
*Sirenintermedia*						643				643	643
REPTILIA	1 317	2 637	563	4 517	495	7 162	907	44	189	8 797	13 314
Crocodylia	321	1	174	496		5				5	501
Alligatoridae	321	1	174	496		5				5	501
*Alligatormississippiensis*	321	1	174	496		5				5	501
Squamata	704	2 504	227	3 435	476	6 403	889	41	175	7 984	11 419
Sauria	508	1 910	216	2 634	424	3 105	773	21	171	4 494	7 128
Anguidae	9	1 107	1	1 117	13	101	18	3	1	136	1 253
*Ophisaurusattenuatus*	9	1 107	1	1 117	13	101	18	3	1	136	1 253
Crotaphytidae						1				1	1
*Crotaphytuscollaris*						1				1	1
Dactyloidae	455	139	145	739	3	201				204	943
*Anoliscarolinensis*	53	30	19	102	3	191				194	296
*Anolissagrei*	402	109	126	637		10				10	647
Eublepharidae						2				2	2
*Coleonyxbrevis*						2				2	2
Gekkonidae	7	38	3	48	2	220	2			224	272
*Hemidactylusmabouia*						4				4	4
*Hemidactylusturcicus*	7	38	3	48	2	216	2			220	268
Iguanidae						5				5	5
*Ctenosauraacanthura*						1				1	1
*Ctenosaurapectinata*						3				3	3
*Ctenosaurasimilis*						1				1	1
Phrynosomatidae	14	509	53	576	385	1 816	718	14	155	3 088	3 664
*Cophosaurustexanus*						2				2	2
*Holbrookiapropinqua*	13	509	49	571	379	966	717	10	154	2 226	2 797
*Holbrookiasubcaudalis*						75				75	75
*Phrynosomacornutum*			1	1	6	180	1	2		189	190
*Sceloporusconsobrinus*						117				117	117
*Sceloporuscyanogenys*						5				5	5
*Sceloporusgrammicus*						195				195	195
*Sceloporusolivaceus*	1		3	4		139		1	1	141	145
*Sceloporusvariabilis*						136		1		137	137
*Urosaurusornatus*						1				1	1
Scincidae	2	37	2	41	2	268	11		1	282	323
*Plestiodonobsoletus*	1	6		7		54	7			61	68
*Plestiodontetragrammus*			2	2		132			1	133	135
*Scincellalateralis*	1	31		32	2	82	4			88	120
Teiidae	21	80	12	113	19	491	24	4	14	552	665
*Aspidoscelisgularis*			7	7	1	380			6	387	394
*Aspidoscelislaredoensis*						1				1	1
*Aspidoscelissexlineatus*	21	80	5	106	18	110	24	4	8	156	262
Serpentes	196	594	11	801	52	3 298	116	20	4	3 490	4 291
Colubridae	166	584	9	759	45	2 594	94	19	4	2 756	3 515
Arizonaelegans	7	6	1	14	6	41	8		1	56	70
*Cemophoralineri*		1		1		12	1			13	14
*Coluberconstrictor*		11		11		90	2			92	103
*Coniophanesimperialis*						64				64	64
*Diadophispunctatus*	1			1							1
*Drymarchonmelanurus*						83				83	83
*Drymobiusmargaritiferus*						18				18	18
*Faranciaabacura*						3				3	3
*Ficimiastreckeri*						11				11	11
*Haldeastriatula*						43				43	43
*Heterodonkennerlyi*						3				3	3
*Heterodonplatirhinos*		3		3	3	15	8			26	29
*Hypsiglenajani*						7				7	7
*Lampropeltisannulata*		5		5	4	37	15			56	61
*Lampropeltiscalligaster*						10				10	10
*Lampropeltisgentilis*	2			2							2
*Lampropeltisgetulacomplex*						13		1		14	14
*Lampropeltisholbrooki*	14			14	2	24				26	40
*Lampropeltissplendida*	2			2		12				12	14
*Leptodeiraseptentrionalis*						13				13	13
*Masticophisflagellum*	25	61	5	91	12	133	25		3	173	264
*Masticophisschotti*						122				122	122
*Nerodiaclarkii*	43	1		44	3	45		16		64	108
*Nerodiacyclopion*						5				5	5
*Nerodiaerythrogaster*						20				20	20
*Nerodiafasciata*						3				3	3
*Nerodiarhombifer*		4		4		163	2			165	169
*Opheodrysaestivus*						56				56	56
*Pantherophisemoryi*	19	19		38		161	4			165	203
*Pantherophisobsoletus*	1			1		17				17	18
*Pituophiscatenifer*		4		4		79				79	83
*Reginagrahami*						7				7	7
*Rhinocheiluslecontei*						10				10	10
*Salvadoragrahamiae*			2	2		70				70	72
*Sonorasemiannulata*						51				51	51
*Storeriadekayi*	1	13	1	15		188	1			189	204
*Tantillagracilis*		9		9		52	3			55	64
*Tantillanigriceps*						49				49	49
*Thamnophismarcianus*	10	33		43	14	341	8	1		364	407
*Thamnophisproximus*	41	413		454	1	519	17			537	991
*Thamnophissirtalis*						4				4	4
*Tropidoclonionlineatum*		1		1				1		1	2
Elapidae						113				113	113
*Micrurustener*						113				113	113
*Leptotyphlopidae*	1			1		102				102	103
*Renadulcis*	1	1		1		102				102	103
*Pythonidae*						1				1	1
*Pythonregius*						1				1	1
Typhlopidae	1		1	2		6				6	8
*Indotyphlopsbraminus*	1		1	2		6				6	8
Viperidae	28	10	1	39	7	482	22	1		512	551
*Agkistrodoncontortrix*						3				3	3
*Agkistrodonpiscivorus*						106		1		107	107
*Crotalusatrox*	28	7	1	36	7	363	3			373	409
*Sistrurustergeminus*		3		3		10	19			29	32
Testudines	292	132	162	586	19	754	18	3	14	808	1 394
Cheloniidae	88	64	35	187	11	1	12		11	35	222
*Carettacaretta*	2	10	1	13	3		2		1	6	19
*Cheloniamydas*	81	26	23	130	2	1	4		8	15	145
*Eretmochelysimbricata*		2	1	3	4		2		1	7	10
*Lepidochelyskempii*	5	26	10	41	2		4		1	7	48
Chelydridae	1	1		2							2
*Chelydraserpentina*	1	1		2							2
Dermochelyidae			1	1			3			3	4
*Dermochelyscoriacea*			1	1			3			3	4
Emydidae	187	65	109	361	1	405	3	3	2	414	775
*Malaclemysterrapin*						34			2	36	36
*Pseudemysnelsoni*			7	7							7
*Terrapenecarolina*	2			2		3				3	5
*Terrapeneornata*	3			3		29		2		31	34
*Trachemysscripta*						8				8	8
*Trachemysscriptaelegans*	182	65	95	342	1	331	3	1		336	678
*Trachemysscriptascripta*			7	7							7
Kinosternidae	15	2		17	7	80				87	104
*Kinosternonflavescens*	15	2		17	7	80				87	104
Testudinidae						212			1	213	213
*Gopherusberlandieri*						212			1	213	213
Trionychidae	1		17	18		56				56	74
*Apalonespinifera*	1		17	18		56				56	74
**Total Amphibians and Reptiles**	1 461	2 843	600	4 904	639	12 951	971	47	222	14 830	19 734

Eleven amphibian and 36 reptile species occur or have occurred naturally or as introduced or accidental species on North Padre Island (Table 2; Suppl. material 1). Of those, eight species of amphibians and 27 species of reptiles are represented or were represented by specimens in natural history collections. Three species of amphibians and nine species of reptiles that occur on North Padre Island are known only from iNaturalist observations; one of those amphibians is known only from an audio file. Two species of reptiles are known only from museum specimens.

Nine amphibian and 39 reptile species currently occur or historically occurred on Mustang Island. Twenty-one reptile species and four amphibian species occur or have occurred on SPI. In all, there are 47 species of reptiles and 12 species of amphibians that occur or have occurred naturally or as introduced or accidental species on North and South Padre and Mustang islands (hereafter “the South Texas barrier islands” or “the islands”).

By comparison, 31 amphibian species and 93 reptile species occur in the seven counties that include or are adjacent to the barrier islands. Eleven species of amphibians and 22 species of reptiles that occur on the mainland, do not occur on the islands. Seven species of reptiles and one amphibian species that occur on Mustang Island do not occur on the other barrier islands. Five amphibian species and seven reptile species that occur on North Padre Island do not occur on the other islands. Three amphibian species and six reptile species that occur on South Padre Island are not known from the other barrier islands.

Six species recorded during the 2002–2003 surveys were first records of those species from North Padre Island: *Pseudacrisclarkii* (spotted chorus frog), *Bufowoodhousii* (Woodhouse’s toad), *Cemophoralineri* (Texas scarletsnake), *Pantherophisemoryi* (Great Plains ratsnake), *Gastrophrynecarolinensis* (eastern narrow-mouthed toad), and *Hylasquirella* (squirrel tree frog). Subsequently, in coordination with staff at PINS, I confirmed four more species not previously known from North Padre Island: *Ranasphenocephala* (southern leopard frog), *Chelydraserpentina* (common snapping turtle), *Renadulcis* (plains threadsnake), and *Bufonebulifer* (gulf coast toad). The records for *R.sphenocephala*, *B.woodhousii, G.carolinensis*, and *C.serpentina* were the first records for Kleberg County (Seabury et al. 2005; Duran and Hall 2013; Walker 2019). *Cemophoralineri* and *Heterodonplatirhinos* (eastern hognose snake) have not been observed on the island since our team captured specimens in 2002.

### Species accounts

The species accounts that follow characterize and enumerate records for each current, past, or potential species or subspecies that occur, possibly occur, or were previously thought to occur, on Padre and Mustang islands. Specimens contained in museum collections, iNaturalist observations, and records based on verifiable photos are included. Audio recordings are included as Suppl. materials.

Results from this inventory (2002–2021) are compared to a checklist of amphibians and reptiles of PINS (only PINS; Rabalais (1975) and a checklist of the herpetofauna of North Padre Island and Mustang Island by Baker and Rabalais (1978). For some species, a short account adequately describes the significance of its presence on the island, while a lengthier account is presented to address the unique nature of some occurrences. I discuss ecological associations for specimens we captured or observed, which is not meant to imply that those species do not occur in other ecological associations. For all mentions of iNaturalist, the citation is iNaturalist.org (accessed 08 October 2020). For unique iNaturalist observations an URL is included, and links to all iNaturalist records from the islands are available in Suppl. material 1.

### Class Amphibia

**Order Anura**

**Family Bufonidae**

#### Bufo nebulifer

Taxon classificationAnimaliaAnuraBufonidae

Girard, 1854

B0E5DBAF-5E9C-5EA1-9568-FC2BF0B21A51

**Figure 4.**
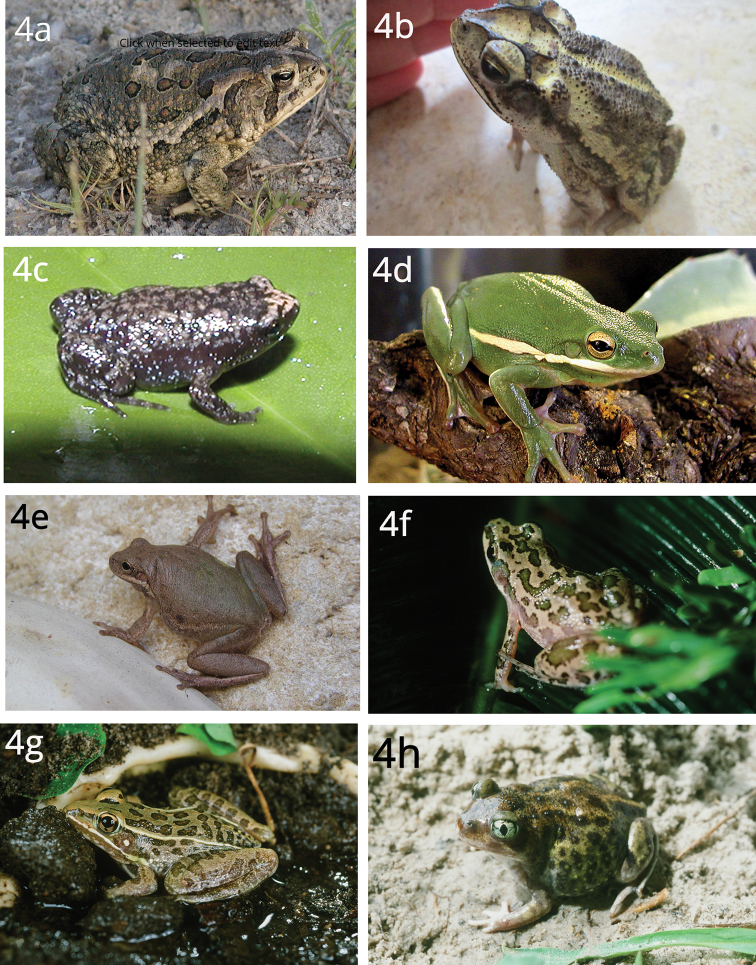
Eight Anurans found on North Padre Island **a***Bufowoodhousii* (Woodhouse’s toad) **b***Bufonebulifer* (Gulf Coast toad; photograph by Alicia Walker) **c***Gastrophrynecarolinensis* (eastern narrow-mouthed toad) **d***Hylacinerea* (Green treefrog) **e***Hylasquirella* (squirrel treefrog) **f***Pseudacrisclarkii* (spotted chorus frog) **g***Ranaberlandieri* (Rio Grande leopard frog) **h***Scaphiopushurterii* (Hurter’s spadefoot).

Gulf Coast toad Fig. 4a

##### Notes.

There are six museum specimens of *B.nebulifer* from NPI, with imprecise locality information, collected in 1891 (USNM 45349–52 and 46150). Our team did not detect *B.nebulifer* during the 2002–2003 surveys. Baker and Rabalais (1978) reported that *B.nebulifer* was not known from NPI but that it was known from residential areas of Mustang Island. A *Bufonebulifer* photographed in 2007 (photo voucher, TNHC 101563) by a PINS biologist became the first verifiable record in over a century; however, in recent years the *B.nebulifer* population on the NPI has gone from nearly undetectable to relatively common as 18 iNaturalist observations were posted 2017–2021. There have been 46 observations posted on iNaturalist from Mustang Island in recent years and 18 from SPI. I located 386 specimens from the seven counties, six specimens, all collected in the 1970s, from Mustang Island, and one specimen from South Padre Island, collected in 1891. Moore (1976) reported that *B.nebulifer* was present on Mustang Island during his 1971 study, but that it was less common than *B.speciosus* and that he did not observe evidence of *B.nebulifer* reproduction.

*Bufonebulifer* breeds in ephemeral pools and wetlands but may be found in much drier habitats and under artificial lighting. The one specimen photographed during this inventory was found in the parking lot of the Malaquite Visitor Center within PINS.

#### Bufo speciosus

Taxon classificationAnimaliaAnuraBufonidae

Girard, 1854

203B6D9C-1FA9-5CD6-8C52-18B1D0C32994

##### Notes.

Baker and Rabalais (1978) reported that *B.speciosus* had been collected on the north end of NPI, but I found no other evidence that *B.speciosus* occurs or has occurred there or on SPI. Baker and Rabalais (1978) also reported that *B.speciosus* was abundant on Mustang Island, which the museum record supports: I located 121 specimens from Mustang Island, including 51 specimens collected on one night in 1968 and 24 specimens collected on one night in 1954. I did not find any specimens collected after 1968 from Mustang Island; however, Moore (1976) reported that *B.speciosus* was the most common anuran he observed during his 1971 study on Mustang Island. I found 580 specimens from the seven counties. It appears that *B.speciosus* has been extirpated from Mustang Island.

#### Bufo woodhousii

Taxon classificationAnimaliaAnuraBufonidae

Girard, 1854

DC81C2A2-EDCA-516C-866E-0470A8CA2D05

Woodhouse’s toad Fig. 4b

##### Notes.

The first record for *B.woodhousii* from NPI and Kleberg County was collected during the 2002–2003 surveys. Another specimen collected in 2004 was the basis for a Kleberg County record published by Seabury et al. (2005). The species was not mentioned by Rabalais (1975) or Baker and Rabalais (1978). The species is not known from Mustang Island or from the counties adjacent to Mustang Island. After our observations of *B.woodhousii* on NPI in 2002, this species was not recorded there again until an observation was posted to iNaturalist in September 2018.

Three specimens from Mustang Island in the TCWC collection were identified as hybrids of *B.woodhousii* and *B.speciosus* (TCWC 93746-48) by the collector (J. K. Baker). I examined those specimens and observed that two of the specimens have weak cranial crests, which are rarely, and then only indistinctly, present in *B.speciosus* but always present in *B.woodhousii*; one of the specimens appears to have a faint mid-dorsal stripe, which is rarely and then only weakly present in *B.speciosus*, but usually present in *B.woodhousii*. While these characteristics provide some morphological support for Baker’s identification, the specimens fall within the range of variation for *B.speciosus*. Because *B.woodhousii* is not known from Mustang Island, hybrids are unlikely, but it is possible that *B.woodhousii* was extirpated before any observations were recorded.

Dixon (2013) reported that a disjunct population of *B.woodhousii* is known on the mainland from the southern Texas counties of Brooks, Cameron, Hidalgo, Kenedy, and Willacy. I found nine museum specimens from Brooks (1), Cameron (3), Hidalgo (1), and Kenedy (4) counties (Table 3). It seems likely that *B.woodhousii* occurs or occurred in the Sand Sheet portion of northern Willacy County, but I was unable to find a specimen or other verifiable record from that county. The species was not thought to occur in Willacy County by Brown (1950), Axtell (1963), or Raun and Gehlbach (1970). Tipton et al. (2012) and Dixon (2013) reported that the species may have been extirpated from the inland portion of the southern Texas counties, but an iNaturalist observation of an individual observed in Kenedy County on 29 October 2015 and submitted in 2018, became the first verifiable South Texas mainland record since 1975 (https://www.inaturalist.org/observations/13049736). During the 2002–2003 surveys, *Bufowoodhousii* was observed or captured mostly in emergent and ephemeral wetlands but was also observed and captured in dry and sparsely vegetated foredunes and in an asphalt parking lot.

**Table 3.** All known mainland occurrences of *Bufowoodhousii* in South Texas.

Inst Code	Cat #	Scientific Name	Date	County	Locality	Collector/observer
BUMMC	R 19809	Bufowoodhousii	10.9.1941	Kenedy	Armstrong	Brown, Bryce C.
TCWC	20830	Bufowoodhousii	11.4.1965	Brooks	12 mi SW Falfurrias	THS Field Meet
AMNH	A174386	Bufowoodhousii	2.7.1966	Kenedy	4.5 mi S Riviera	Ernest A. Liner & L.D. Wilson
AMNH	A183140	Bufowoodhousii	8.4.1968	Kenedy	3.4 mi N Armstrong on US Hwy 77	Allan H. Chaney
AMNH	A183141	Bufowoodhousii	8.4.1968	Kenedy	3.4 mi N Armstrong on US Hwy 77	Allan H. Chaney
AMNH	A183142	Bufowoodhousii	8.4.1968	Kenedy	3.4 mi N Armstrong on US Hwy 77	Allan H. Chaney
UTA	8382	Bufowoodhousii	8.8.1975	Cameron	Near San Benito	1, J. L. Darling
UTA	8383	Bufowoodhousii	8.8.1975	Cameron	Near San Benito	1, J. L. Darling
INAT	13049736	Bufowoodhousii	29.10.2015	Kenedy	Kenedy County (obscured)	Bryan Calk
KU	294840	Bufowoodhousii	na	Cameron	Brownsville	J.C. Lee

###### Family Microhylidae

#### Gastrophryne carolinensis

Taxon classificationAnimaliaAnuraMicrohylidae

Holbrook, 1835

63C1212B-E155-58DD-ABA2-36C8F9CD3820

Eastern narrow-mouthed toad Fig. 4c ; Suppl. material 2

##### Notes.

During the 2002–2003 surveys, I made several audio recordings of *G.carolinensis* (Suppl. material 2; iNaturalist observation https://www.inaturalist.org/observations/12052215), which were the first verifiable records for that species from Kleberg County or from the South Texas barrier islands. I collected three *Gastrophryne* tadpoles from a seasonally inundated wetland and raised them until shortly after metamorphosis, when they could be identified as *G.carolinensis*. I photographed them (Fig. 4c), but a predator took the specimens before they could be preserved. Two museum specimens (TNHC 96013, 96014) collected in emergent wetlands in 2015 by PINS staff member Alicia Walker were the first specimens of *G.carolinensis* from Kleberg County and the South Texas barrier islands (Walker 2019). Five audio recordings of *G.carolinensis* were posted to iNaturalist in 2017. All specimens were collected or observed in emergent wetlands and ephemeral pools within the grassland/wetland matrix.

#### Gastrophryne olivacea

Taxon classificationAnimaliaAnuraMicrohylidae

Hallowell, 1856

29674DC4-9949-5043-8C56-A378239283F5

##### Notes.

There were no records for *G.olivacea* for the South Texas barrier islands until a photo was posted on iNaturalist in 2017 (https://www.inaturalist.org/observations/3447802; photo voucher TNHC 112542). I could not confirm the identification with certainty, but the observer describes characteristics not shown in the photo that would confirm the identification (T. LaDuc, pers. comm.). In March 2020, the first observation of *G.olivacea* from Mustang Island was entered on iNaturalist (https://www.inaturalist.org/observations/40571319). The animal shown is not a typical *G.olivacea* but neither is it typical *G.carolinensis*. I located 316 museum specimens from the mainland portion of the seven counties. Rabalais (1975) characterized the species as possible for PINS.

#### Family Eleutherodactylidae

##### Eleutherodactylus campi

Taxon classificationAnimaliaAnuraEleutherodactylidae

Stejneger, 1915

85FB83A6-459C-5772-A324-F196CFBCB6A7

###### Notes.

This species was once found mostly in northeastern Mexico and in the Rio Grande Valley of Texas but has expanded its range to include isolated populations in southern Louisiana and most of the eastern third of Texas to the Red River. It is often found in plants and other items transported from the Rio Grande Valley (AmphibiaWeb 2021). Our team did not detect *E.campi* on NPI and there are no other records, but there are three iNaturalist observations from Mustang Island and two from SPI. There are three museum records from SPI and 184 museum records from the mainland portion of the seven counties. It seems likely that *E.campi* will eventually show up on NPI or that it is already there and has not yet been reported.

##### Eleutherodactylus planirostris

Taxon classificationAnimaliaAnuraEleutherodactylidae

Cope, 1862

E41FCFE1-B274-57E0-B4A1-CA84EE2C05B4

###### Notes.

*Eleutherodactylusplanirostris* is native to Cuba, the Bahamas, the Cayman Islands, and the Turk and Caicos Islands (AmphibiaWeb 2021) but is now found in coastal areas around the Gulf of Mexico from Florida to the Yucatan Peninsula of Mexico. There are two museum records from SPI. The same records were posted on iNaturalist: https://www.inaturalist.org/observations/62578832 and https://www.inaturalist.org/observations/62578829). The species has not been recorded in the rest of the study area.

##### Osteopilus septentrionalis

Taxon classificationAnimaliaAnuraEleutherodactylidae

Duméril & Bibron, 1841

3DF689E4-10DD-5E14-9314-90AFDC72017B

###### Notes.

*Osteopilusseptentrionalis* is native to the Bahamas, Cayman Islands and Cuba and has been introduced to other Caribbean Islands, most of Florida, and is spottily distributed in coastal areas from Georgia to Texas (AmphibiaWeb 2021). There is only one record for the species from the study area, an iNaturalist observation from Port Isabel in Cameron County. It was not found on the islands but is included here because of its potential to disrupt invaded ecosystems. It preys on a variety of animals including other amphibians and reptiles. In urban areas in Florida, *O.septentrionalis*, by predation and competition, has displaced some native frogs including (but not limited to) *Hylacinerea* (green treefrogs) and *H.squirella* (squirrel treefrog). The University of Florida Department of Wildlife Ecology recommends capturing and humanely euthanizing any *O.septentrionalis* found (Johnson 2017).

#### Family Hylidae

##### Hyla cinerea

Taxon classificationAnimaliaAnuraHylidae

Schneider, 1799

023DC9FD-CBFE-5F3F-BA6E-16F643BD570B

Green treefrog Fig. 4d

###### Notes.

During and after rains during warmer months in 2002 and 2003 our team recorded conspicuous choruses of *H.cinerea* at all calling-frog stations and collected one of the 14 museum specimens from NPI. The historical record indicates that the species is considerably less common in other years. Rabalais (1975) characterized the species as “uncommon.” I located 474 specimens from the mainland portion of the seven counties but only one specimen and nine iNaturalist observations from Mustang Island and no records from South Padre Island. Most observations during this inventory were in emergent wetlands during the breeding season.

The status of this species in southern portion of the study area is unclear. I heard choruses of *H.cinerea* at The Nature Conservancy’s Southmost Preserve, in Cameron County, in 2002. There are ten museum specimens from Cameron County but no iNaturalist records. There are 34 museum specimens and zero iNaturalist observations from Kenedy County. The newest specimen from Cameron County was collected in 1941, and the newest specimen from Kenedy County was collected in 1976. The only record of any kind from the mainland portion of the counties south of Nueces County is an iNaturalist audio recording made in 2015 near Port Mansfield in Willacy County: https://www.inaturalist.org/observations/1480951. The status of the species in southern Texas deserves further study.

##### Hyla squirella

Taxon classificationAnimaliaAnuraHylidae

Bosc, 1800

81F80A43-3188-5FFF-9B6F-7CE49022345F

Squirrel treefrog Fig. 4e ; Suppl. material 3

###### Notes.

There were no records for *H.squirella* from NPI until I photographed and audio recorded it during the 2002–2003 surveys. The calls were recorded in emergent wetlands during breeding season (Suppl. material 3; iNaturalist Observation https://www.inaturalist.org/observations/49370292), but I was unable to collect a specimen during those early surveys. There are two museum records from Mustang Island and 28 from South Padre islands but none from NPI. Sixty-six iNaturalist observations from NPI, 55 from Mustang Island, and ten from SPI based on photos and calls, were posted from 2015 to 2020. The species was not mentioned by Rabalais (1975) or by Baker and Rabalais (1978).

There is still only one mainland museum record collected from the study area south of Kleberg County of a specimen taken near Port Mansfield, in Willacy County in 2015. That specimen was also posted to iNaturalist where the collector commented that it was a single individual calling from a roadside ditch. The 28 museum records from Cameron County were all from a small area in the urbanized portion of southern SPI.

##### Pseudacris clarkii

Taxon classificationAnimaliaAnuraHylidae

Baird, 1854

31A9E3A1-B29C-56AF-8624-236F12AD71BD

Spotted chorus frog Fig. 4f

###### Notes.

Our team did not observe or hear *P.clarkii* in 2002, but in 2003 I made audio recordings and collected a specimen (TCWC 93884) in temporarily flooded grasslands during drought-ending tropical rains. That specimen remains the only specimen of *P.clarkii* collected from the South Texas barrier islands, and among the eight research grade iNaturalist observations, the only observation based on a photo (https://www.inaturalist.org/observations/12007443). The species is not known from museum or iNaturalist records from SPI or Mustang islands. Only 18 of the 231 museum specimens from the study area have been collected since 1990. Rabalais (1975) categorized *P.clarkii* as “possible.” Baker and Rabalais (1978) did not mention it.

#### Family Ranidae

##### Rana berlandieri

Taxon classificationAnimaliaAnuraRanidae

Baird, 1859

8F2E06C2-C8F1-51CF-8254-4D56BF61BA87

Rio Grande leopard frog Fig. 4g

###### Notes.

There are seven museum specimens of *R.berlandieri* from NPI and three from Mustang Island residing in the collections I surveyed. I found 584 specimens from the mainland portion of the seven counties. During the 2002–2003 surveys, I collected two specimens and audio-recorded the species at most ephemeral and man-made ponds in the 24 km south of Packery Channel.

I collected one specimen that was particularly noteworthy because of its locality: TCWC 93885 was collected ~ 80.5 km south of the southern end of Park Road 22. That locality is a narrow, arid, and sparsely vegetated part of the island between two wash-over channel depressions that sometimes hold freshwater but are periodically flooded with saline water from the Gulf of Mexico and hypersaline water from the Laguna Madre. Permanent or long-lasting freshwater, usually associated with *R.berlandieri*, is not present. When I last visited that site in June 2018, the ponds only supported halophytic vegetation around its edges, which were crusted with salt. The survival strategies and dynamics of that population segment deserve further study.

##### Rana catesbeiana

Taxon classificationAnimaliaAnuraRanidae

Shaw, 1802

17607BC2-2DA4-5595-8511-6FEEBF72E1FE

###### Notes.

A 2018 iNaturalist observation, based on an audio file, is the first and only record of *Ranacatesbeiana* on NPI (https://www.inaturalist.org/observations/8027494). Other experienced listeners have concurred on the identification of that call, but because of the poor quality of the recording, I cannot confirm with complete certainty that the call is that of *R.catesbeiana*. The NPI record is from the northern tip of the island, near Packery Channel. The species is relatively common in Aransas, Nueces, and San Patricio counties and much of the United States but uncommon in the counties adjacent to Padre Island. There were 77 museum specimens from the seven counties. The species requires relatively permanent freshwater for its long-lived larvae, therefore its habitat on NPI is probably limited to several small man-made ponds within the urbanized northernmost part of the island and three manmade ponds in the northern part of the Padre Island National Seashore. The lake that occurs near the middle of the island, from ~ 22.5 km south of Packery Channel to ~ 27 km south of Packery Channel is periodically dry but, in some years, might hold water long enough for *R.catesbeiana* reproduction. There is little to no habitat for the species in the southern 143 km of Padre Island. The species is a non-native and invasive in the western United States, where it competes with and preys on native fauna. Its recent arrival on North Padre and Mustang islands might be considered invasive.

##### Rana sphenocephala

Taxon classificationAnimaliaAnuraRanidae

Cope, 1886

14952962-912F-5687-8DE9-20BE3189C4EE

Southern leopard frog Suppl. material 4

###### Notes.

There is one *Ranasphenocephala* specimen from Padre Island (TNHC 65562), which was also the first and only specimen from Kleberg County (Duran and Hall 2013). There are no other records for the South Texas barrier islands or from the inland portion of the counties adjacent to Padre Island south of Nueces County. There are 176 museum specimens from the mainland counties adjacent to Mustang Island. In 2013, I made an audio recording of *R.sphenocephala* (Suppl. material 4; iNaturalist observation: https://www.inaturalist.org/observations/314642), which was only the second verifiable record for the islands and the first within PINS. That recording may include *R.berlandieri* calling at the same time, but that is not clear. The species was not mentioned by either Rabalais (1975) or Baker and Rabalais (1978).

Dorsolateral ridges inset posteriorly at the groin in *R.berlandieri* usually distinguish it from *R.sphenocephala*, but dorsolateral ridges of some *Rana* specimens from NPI are not distinctly inset. We examined many specimens in the field and could never say that any of them were distinctly typical of *R.sphenocephala*. While morphological differences between the species were not consistently differentiating, their calls are quite different and perhaps better evidence of their occurrence on the island than specimens or photos. The audio file of *R.sphenocephala* calling may also include *R.berlandieri* calling at the same time. Hillis (1981) reported on sympatric populations of three ranid species and identified pre-mating isolating mechanisms, including staggered breeding times and habitat.

#### Family Scaphiopodidae

##### Scaphiopus couchii

Taxon classificationAnimaliaAnuraScaphiopodidae

Baird, 1854

938D89AB-702A-5DB9-BB69-3DEAC37B72B7

###### Notes.

I did not find verifiable records of *Scaphiopuscouchii* from the South Texas barrier islands. It is mentioned here because Baker and Rabalais (1978) reported that a specimen had been collected “from a freshwater pond area along Park Road 22 north of the entrance to the National Seashore,” but I did not locate that specimen. It may not exist or may have been re-identified. I found 792 *S.couchii* specimens from the mainland portion of the seven counties.

##### Scaphiopus hurterii

Taxon classificationAnimaliaAnuraScaphiopodidae

Strecker, 1910

C94C76B7-EA13-5F6D-8499-8D2845B9F385

Hurter’s spadefoot Fig. 4h

###### Notes.

I located 30 museum specimens from NPI, nine of which were collected during the 2002–2003 surveys. I found five museum specimens from Mustang Island and 235 from the mainland portion of the seven counties. There are 61 iNaturalist observations from NPI, one from Mustang Island and none from South Padre Island. *Scaphiopushurterii* was captured 34 times during the 2002–2003 surveys at four study sites, all in flooded grasslands within grassland/wetland matrices. It was audio-recorded at every stop on the calling-frog survey route. The species is conspicuous during and after rains but nearly undetectable by the casual observer during dryer times.

### Class Reptilia

**Order Testudines**

#### Families Cheloniidae and Dermochelyidae (sea turtles)

**Figure 5.**
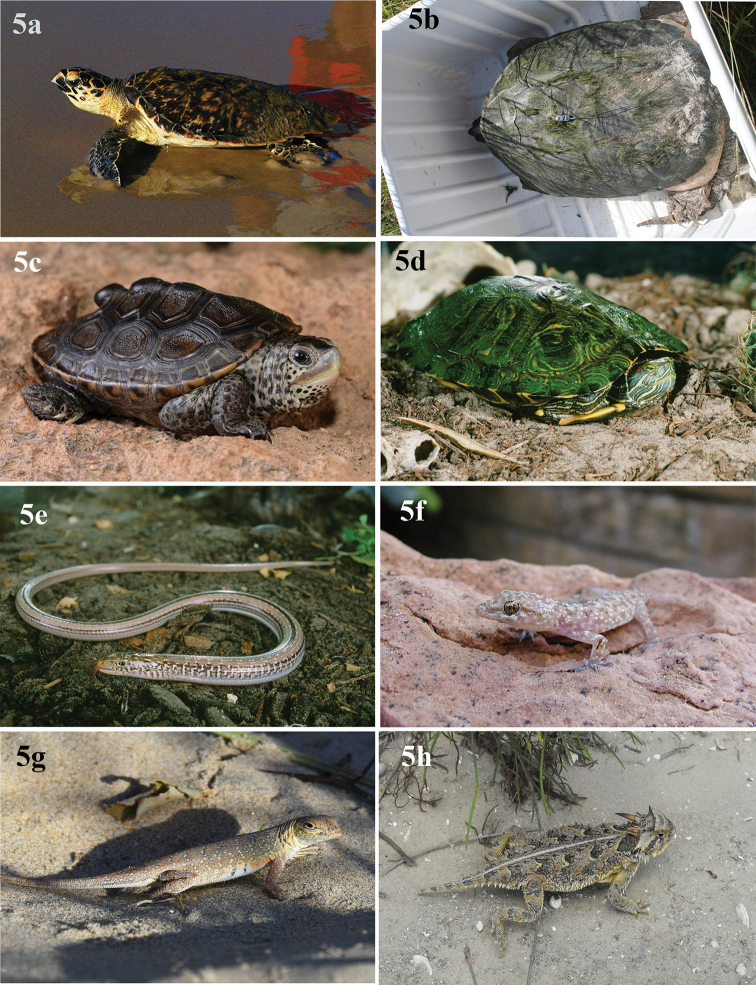
Eight reptiles that occur on North Padre Island **a***Eretmochelysimbricata* (Hawksbill sea turtle) **b***Chelydraserpentina* (common snapping turtle) **c***Malaclemysterrapin* (diamondback terrapin) **d***Trachemysscripta* (pond slider) **e***Ophisaurusattenuatus* (slender glass lizard) **f***Hemidactylusturcicus* (Mediterranean house gecko) **g***Holbrookiapropinqua* (keeled earless lizard) **h***Phrynosomacornutum* (Texas horned lizard; photograph by Jerry Batey).

Padre Island National Seashore Division of Sea Turtle Science and Recovery has conducted a sea turtle monitoring, research, and recovery program since the 1970s (https://www.nps.gov/pais/learn/seaturtles.htm). The program is centered around monitoring and recovery of Kemp’s Ridley Sea Turtle (*Lepidochelyskempii*) but has recorded the presence of four additional species: Loggerhead Sea Turtle (*Carettacaretta*), Green Sea Turtle (*Cheloniamydas*), Leatherback Sea Turtle (*Dermochelyscoriacea*), Hawksbill Sea Turtle (*Eretmochelysimbricata*). Dozens of staff and volunteers patrol the beaches for Kemp’s Ridley nests each summer. Eggs are excavated and hatched in the lab. Hatchling releases, which usually occur from June-August each year, are popular public events. I was not charged with conducting field surveys for sea turtles, but we did occasionally encounter sea turtles on the beach, including the Hawksbill Sea Turtle shown in Figure 5a. Table 4 is a compilation of iNaturalist observations and museum specimens of sea turtles from the islands and surrounding waters, but given the serendipitous nature of sea turtle observations, Table 4 does not accurately represent the true relative abundance of these species.

**Table 4.** Sea turtle iNaturalist and museum records from the South Texas barrier islands. **MI** = Mustang Island; **NPI** = North Padre Island; **SPI** = South Padre Island iNat = iNaturalist observation (accessed 06 Oct 2020); Mus = Museum Specimens (Vertnet.org, accessed 02 Oct 2020).

iNaturalist Observations					Museum Specimen Records					All Totals
	MI	NPI	SPI	iNatTot	MI	ML	NPI	SPI	MusTot	
**Cheloniidae**	**88**	**64**	**35**	**187**	**11**	**1**	**12**	**11**	**35**	**222**
Carettacaretta	2	10	1	13	3		2	1	6	19
Cheloniamydas	81	26	23	130	2	1	4	8	15	145
Eretmochelysimbricata		2	1	3	4		2	1	7	10
Lepidochelyskempii	5	26	10	41	2		4	1	7	48
**Dermochelyidae**			**1**	**1**			**3**		**3**	**4**
Dermochelyscoriacea			1	1			3		3	4

#### Family Chelydridae

##### Chelydra serpentina

Taxon classificationAnimaliaTestudinesChelydridae

Linnaeus, 1758

18E7CEEA-7E2D-56AE-B777-0D662BCBE928

Common snapping turtle Fig. 5b

###### Notes.

A photo taken by a PINS staff member in 2007 (TNHC 86867) is the only verifiable record of *Chelydraserpentina* from the South Texas barrier islands, though Baker and Rabalais (1978) reported that a live specimen had washed up on an NPI beach near the Malaquite Visitor Center. The species is not known from the counties adjacent to North and South Padre Island (Dixon 2013). I witnessed a PINS visitor attempting to release a red-eared slider (*Trachemysscriptaelegans*) into the Gulf of Mexico surf in 2007 and suspect *C.serpentina* may have arrived on the island as a similar misguided rescue attempt. Baker and Rabalais (1978) speculated that freshwater Chelonians might be washed into the Gulf by flooding, then carried out of their native range by longshore currents. There is no evidence that *C.serpentina* occurs on the islands naturally.

#### Family Emydidae

##### Malaclemys terrapin

Taxon classificationAnimaliaTestudinesEmydidae

Schoepff, 1793

F325DBBF-B929-50D9-A8CA-879653660FE8

Diamondback terrapin Fig. 5c

###### Notes.

There were no specimens or iNaturalist observations of *M.terrapin* from the South Texas barrier islands until one juvenile was collected, and another juvenile was photographed in an urbanized part of southernmost South Padre Island in February and March 2019 (Guadiana et al. 2020). The live specimen (photo voucher TNHC 114470; Fig. 5c), which was collected ~200 m west of the forebeach, is now housed in the Gladys Porter Zoo in Brownsville, Texas. The photo of the other specimen (TNHC 114630) appears to have been taken on the wet sand of the forebeach. Those localities are ~ 180 km south of the nearest localities in Corpus Christi Bay. Within the study area, there are 30 museum specimens from estuarine bays and marshes in Aransas, Nueces, and San Patricio counties. There are 80 museum specimens in all of Texas. Salt secreting glands allow *M.terrapin* to adjust to changing salinity, but there are no verifiable records from the hypersaline Laguna Madre, and it is rarely observed in 100% seawater; I found only one iNaturalist record (https://www.inaturalist.org/observations/21335833) and one museum specimen (TNHC 7421) from the seaward side of a Texas barrier island or peninsula: both were found on the Bolivar Peninsula, one near Rollover Pass and one near the mouth of Galveston Bay. Brackish marshes, usually associated with the species, are mostly absent along the shores of the Upper Laguna Madre and sparse along the shores of the Lower Laguna Madre. The species might enter the Upper Laguna Madre during times of lower salinity, then leave as salinity levels rise, a process characterized as “behavioral osmoregulation” by Dunson and Mazzotti (1989). The form that occurs in this part of the range is generally assigned to the subspecies *M.t.littoralis* (Texas diamondback terrapin), but the South Padre Island specimens could not be assigned to subspecies based on morphological characteristics described by Ernst and Lovich (2009) (Drew Davis, pers. comm.). Determining the subspecies by genetic or morphological means might provide a clue about how these animals arrived on SPI so far out of their native range.

##### Terrapene carolina

Taxon classificationAnimaliaTestudinesEmydidae

Linnaeus, 1758

26B44581-0C45-5023-873D-CF6D9464C617

###### Notes.

There were three *T.carolina* museum records from the mainland portion of the study area and two iNaturalist observations from Mustang Island. The mainland occurrences are individual records separated by decades (Suppl. material 1), which suggests they are introductions, probably released pets. While the study area is on the edge of the range for the species, there is no evidence to suggest that it has established reproducing populations there.

##### Terrapene ornata

Taxon classificationAnimaliaTestudinesEmydidae

Agassiz, 1857

9FC264FB-B091-5430-8CB3-4FBD9E5F0AC4

###### Notes.

There are no museum specimens of *T.ornata* from the South Texas barrier islands and no records of any kind from NPI or SPI. Three iNaturalist observations from Port Aransas on Mustang Island have been entered since 2013. Baker and Rabalais (1978) also reported having seen two *Terrapene* sp. road-killed on Mustang Island. I found 24 museum specimens from the adjacent counties. Box turtles are popular pets. In Texas they may be legally taken from the wild for non-commercial purposes. They often escape or are released back into the wild, far from where they were collected.

##### Trachemys scripta

Taxon classificationAnimaliaTestudinesEmydidae

Wied-Neuwied, 1838

692D12F0-5A26-5DF1-B6C6-CE4A0095D1C3

Pond slider Fig. 5d

###### Notes.

*Trachemysscripta* is easily observed on any visit to the three manmade ponds within the Padre Island National Seashore. Our team trapped 24 pond sliders in hoop traps in those ponds. No other turtle species were observed. *Trachemysscripta* was photographed but not collected. I located four museum specimens from NPI, two from Mustang Island, and 287 from the adjacent counties. *Trachemysscripta* from the study area is usually assigned to the subspecies *T.s.elegans* (red-eared slider), but there are seven iNaturalist observations (several of which appear to be the same individual) identified as *T.s.scripta* (yellow-bellied slider) from the South Padre Island Birding and Nature Center at the southern tip of SPI. The yellow-bellied slider is native to the eastern half of the US, but both subspecies have established introduced populations all over the US and Europe.

#### Family Kinosternidae

##### Kinosternon flavescens

Taxon classificationAnimaliaTestudinesKinosternidae

Agassiz, 1857

941FCF21-D761-583F-98C9-12A7412099CB

###### Notes.

Baker and Rabalais (1978) reported that *K.flavescens* was common on Mustang Island and that it had been observed on the northern end of PINS, but the only verifiable records for *K.flavescens* from NPI I found were two iNaturalist observations of road-killed turtles from the northern end of the island entered on 22 and 24 September 2017. The nearest mainland record from those observation is 13.6 km across the Laguna Madre near Oso Bay, and the nearest mainland freshwater habitat for *K.flavescens* is 7.7 km across the Laguna Madre. Because Baker and Rabalais (1978) reported that the species was “common” on Mustang Island, and because there are seven museum records from Mustang Island since 1960 and 15 iNaturalist observations more recently, it is likely that *K.flavescens* is reproducing on Mustang Island. The species is not easily trapped in hoop traps, so our methodology might not have been adequate to determine if *K.flavescens* occurs as a reproducing population on NPI. There are 79 museum specimens from the seven counties and no records from SPI.

#### Family Testudinae

##### Gopherus berlandieri

Taxon classificationAnimaliaTestudinesTestudinae

Agassiz, 1857

90814CA4-9B90-5098-8AE3-35E5AA51C395

###### Notes.

Several occurrences of *Gopherusberlandieri* on the South Texas barrier islands have been recorded, but there is little evidence that suggests the occurrences are natural. One specimen of a *G.berlandieri* (AMNH 9307) appears to have been collected on the southern end of Padre Island in 1917. Baker and Rabalais (1978) found a *G.berlandieri* with a painted carapace dead on the road on Mustang Island and a dead tortoise on the beach on NPI. Baker and Rabalais (1978) also reported that a live tortoise had been found just north of the Mansfield Channel, which divides North and South Padre islands; it is not clear whether they observed that animal themselves or if that was an anecdotal record. I did not find those specimens in natural history collections. Judd and Rose (2000) speculated that *G.berlandieri* populations occur on the barrier islands but offered no evidence of naturally occurring populations. In 2002, Frank Judd told me that he once found a live *G.berlandieri* on SPI but believed that was a human-aided occurrence and did not believe that tortoises occur on SPI naturally (Frank Judd, pers. comm.).

Little habitat for *G.berlandieri* is present on the South Texas barrier islands, and I could find only two reports of naturally occurring tortoises on deep sand: Neill (1958; citing pers. comm. with JR Dixon) reported a tortoise from “sand dunes” near Port Isabel on the mainland, and an iNaturalist observation posted in 2021 appears to show tortoise tracks on mainland sand dunes, also near Port Isabel (https://www.inaturalist.org/observations/74145383). While *G.berlandieri* often occurs on soils with a moderate sand content in the upper soil horizon, it is usually found on moderately clayey or loamy soils (Bury and Smith 1986; Kazmeier et al. 2001). Habitat of *G.berlandieri* at the Atascosa National Wildlife Refuge, adjacent to the Laguna Madre in Cameron County, was described by Bury and Smith (1986) as “lomas” (clay dunes). There is some evidence that the composition of vegetation on the islands also may not be suitable for tortoises, e.g., Scalise (2011) found cactus in 98% of scat of *G.berlandieri* that represented 29.8% of their diet. Cacti (mostly *Opuntia* sp.) occur on the barrier islands but not in the densities usually associated with *G.berlandieri* (Scalise 2011). Habitat for *G.berlandieri* appears to be limited on the South Texas barrier island and there is no evidence of reproducing populations, but the re-appearance of single individuals, probably via human-aided transport, is likely to continue to occur.

### Class Reptilia

**Order Crocodilia**

**Family Alligatoridae**

#### Alligator mississippiensis

Taxon classificationAnimaliaCrocodiliaAlligatoridae

Daudin, 1802

340D5BDC-6D0B-5D35-A2B7-88D418ED5693

##### Notes.

There are no museum records from the South Texas barrier islands and only two from the seven counties, but the museum database is not a good indicator of the abundance an animal whose adult length may be 1.8–5 m (Conant and Collins 1998). While the species is relatively common in the southern and southeastern United States, it is listed by the U.S. Fish and Wildlife Service as threatened by similarity of appearance to rare crocodilians. There is no evidence that alligators have occurred on NPI naturally, but adult alligators have appeared there several times over the years, accidentally or via human introduction; it would not be surprising if it appeared there as an accidental visitor again.

Just prior to the 2002–2003 surveys, NPS had introduced three *A.mississippiensis* to a manmade pond within PINS according to the Natural Resource Manager at the time (Darrel Echols, pers. comm.). In consultation with experts, NPS later determined that alligators had probably never occurred naturally on the island and removed them. In 2007 an individual was found on Big Shell Beach, ~ 40 km south of the southern end of Park Road 22. That animal had been tagged ~ 500 km away, across the Gulf of Mexico at a national wildlife refuge in Louisiana (Buzz Botts, National Park Service, pers, comm.). I added that observation to iNaturalist (https://www.inaturalist.org/observations/20228712), which is the only iNaturalist observation on NPI. Then in 2021, PINS reported on their Facebook page that another alligator washed up on NPI which had also been tagged in Louisiana https://www.facebook.com/FriendsPINS/posts/173996127976128). Those occurrences provide support for the hypothesis that Gulf of Mexico currents play a role in the transport of out-of-range species to the South Texas barrier islands. Alligators will survive in manmade ponds for years, but natural populations require marshy habitat with access to deeper fresh or brackish water (Joanen and McNease 1972). Those habitat types are not found on NPI or the mainland bordering the Upper Laguna Madre, but they are found to the north and south where the species occurs in Nueces and Cameron counties. In Kleberg County, alligators are found within and around the city of Kingsville where they were probably introduced. They are not found west of NPI and the Laguna Madre in Baffin Bay. The Cameron County population is separated from the main alligator population by ~ 125 km of unsuitable habitat, which raises a question about their natural occurrence there, but alligators were first reported from Cameron County by Baird (1859) based on a report by US army officer Stewart Van Vliet, probably around the time of the Battle of Resaca de Palma (on 9 May 1846), during the Mexican-American War. There are 321 iNaturalist observations from the water in and around Mustang Island, and 174 iNaturalist observations from SPI, in or around the Laguna Atascosa Federal Wildlife Refuge. The observations from Mustang and SPI probably include multiple observations of the same individuals.

#### Order Squamata

#### Suborder Sauria

#### Family Anguidae

##### Ophisaurus attenuatus

Taxon classificationAnimaliaSquamataAnguidae

Cope, 1880

348AAB5B-5D49-5A8D-925D-DD7DA3E0CC27

Slender glass lizard Fig. 5e

###### Notes.

*Ophisaurusattenuatus* is one of the most frequently observed reptiles on NPI. I located 17 museum specimens and 1012 iNaturalist observations. During the 2002–2003 surveys, the species was trapped 14 times at all eight study sites between the north end of PINS and the 35-mile-marker. A phenomenon resembling a mass movement of glass lizards was reported on iNaturalist from 24 February to 20 June 2017, when Jon McIntire of Corpus Christi, Texas, reported 938 observations, mostly on Park Road 22 and Bird Island Basin Road on NPI. He reported that the surrounding grasslands had recently been control-burned. The species is less frequently seen on Mustang Island, with five iNaturalist observations and 16 museum specimens. There is only one iNaturalist record and no museum records for the species on SPI; potential observers on SPI rarely venture into the island far from the beach, so the species is probably more common on SPI than that single record might imply. There were 91 museum records from the mainland portion of the seven counties.

#### Family Gekkonidae

##### Hemidactylus turcicus

Taxon classificationAnimaliaSquamataGekkonidae

Linnaeus, 1758

36E92442-F532-5979-A7A0-52546B1097F6

Mediterranean house gecko Fig. 5f

###### Notes.

*Hemidactylusturcicus* is an introduced species native to the Mediterranean region. The earliest specimens I found from Texas were collected in Cameron County in 1953 (TNHC 23057–23060 It is now common on and around manmade structures on the South Texas barrier islands and across the southern United States. The only museum specimens of *H.turcicus* from NPI were collected in 1980 and 1982 (TCWC 93823 and 93845). We did not observe the species during the 2002–2003 surveys, but between 2017 and 2020, 38 iNaturalist observations from NPI were entered; all but two of those were observed in the northernmost residential part of the island. There are four museum specimens and seven iNaturalist observations from Mustang Island. I found 200 museum specimens from the mainland portion of the seven counties.

#### Family Phrynosomatidae

##### Holbrookia propinqua

Taxon classificationAnimaliaSquamataPhrynosomatidae

Baird & Girard 1852

C3FCA7EE-D608-5234-BCEC-1E3C6D5B0844

Keeled earless lizard Fig. 5g

###### Notes.

*Holbrookiapropinqua* is probably the most abundant reptile on the South Texas barrier islands, certainly the most observable. I located 724 museum specimens from NPI and 1916 specimens from the seven counties. During the 2002–2003 surveys, it was trapped 93 times. Another 128 observations were recorded, and hundreds of casual observations by the survey team were not recorded. On NPI, it is most abundant in the back beach/foredunes ecological zone, but it is common on deep dry sand throughout the island. A primary component of *H.propinqua* habitat is deep sand, which is the primary soil component on the islands and of an ~ 800,000 ha area that extends westward from the Land Cut and includes parts of several counties in southern Texas. That area, commonly known as the Sand Sheet, has been altered by grazing, farming, and invasive, nonnative, grasses, mostly Kleberg bluestem (*Dichanthiumannulatum*) and buffelgrass (*Pennisetumciliare*), which has led to its decline on the mainland.

##### Holbrookia subcaudalis

Taxon classificationAnimaliaSquamataPhrynosomatidae

Cope, 1880

12E02775-4033-5BF4-8B3B-7DCDF8575E87

###### Notes.

The catalogue of reptiles and amphibians for the collection kept at Texas A&M, Kingsville (TAMUK) contained an entry for H. (lacerata) subcaudalis (TAMUK 1879) collected in 1968 from the “Dunn Ranch” on NPI, but that specimen was missing when I examined the collection in 2002 and missing when the collection was transferred to AMNH in 2005. Prior to the creation of PINS, most of NPI was part of the Dunn Ranch. When PINS staff speak of the Dunn Ranch, they are generally referring to one of several sites where historical ruins remain. According to the collectors of the TAMUK specimen, the locality was probably the Green Hill site, ~ 1.3 km SW of the 25-mile marker (Thomas Shirley, pers. comm.) although it is possible that they were referring to the Black Hills site, ~ 1.5 km southwest of the 10-mile marker. Our team did not observe the lizard during a nonrandom visual encounter search at the southernmost site and did not trap it in a pitfall array ~ 2 km north of the Green Hill site. I conducted walking surveys at the site six times in subsequent years.

The questionable Dunn Ranch specimen was the only specimen of that species from the South Texas barrier islands. Axtell (1998) commented that the locality or identification of that specimen was probably erroneous. The species is known from just across the Laguna Madre in Kleberg County, along the clayey shores of Baffin Bay, but it avoids deep sand, so it is not likely to occur on NPI.

Both *H.lacerata* and *H.subcaudalis* have been the focus of a considerable amount of survey work and research following a 2011 ruling by the U. S. Fish and Wildlife Service that a listing of threatened or endangered, pursuant to the Endangered Species Act of 1973, may be warranted.

##### Phrynosoma cornutum

Taxon classificationAnimaliaSquamataPhrynosomatidae

Harlan, 1825

E5E51578-9083-55F5-A1D5-7C1BACAD976B

Texas horned lizard Fig. 5h

###### Notes.

Rabalais (1975) listed *P.cornutum* as “possible” for PINS and Baker and Rabalais (1978) reported that it was “common” on Mustang and northern Padre Island in the 1950s and 1960s. I found seven museum specimens from Mustang Island; the most recent of those was 1967. I found one specimen collected on NPI in 1967 but no iNaturalist records. I located 126 museum specimens from the inland portion of the seven counties; of those, 108 had collection dates, and only 15 of the specimens with dates were collected after 1970, and only two were collected since 1987. There are five museum specimens and possibly an obscured iNaturalist record from a small natural island in Corpus Christi Bay. I received a photo of a horned lizard from that island taken in 2013 and have since received other anecdotal accounts of recent observations of *P.cornutum* on that island. *Phrynosomacornutum* has apparently been extirpated from the South Texas barrier islands. It is listed as a threatened species in Texas.

#### Subfamily Sceloporinae

Four species of Sceloporine lizards are known from the mainland portion of the seven counties: *Sceloporusconsobrinus* (prairie lizard), *S.olivaceus* (Texas spiny lizard), *S.variabilis* (rosebellied lizard), and *S.cyanogenys* (blue spiny lizard). No Sceloporine lizards are known from NPI or Mustang Island, but a specimen of *S.olivaceus* was collected on South Padre Island in 1916 (AMNH 8159), and two iNaturalist observations from the urbanized portion of southernmost South Padre Island were entered in March and May of 2020. Sceloporine lizards are unlikely to occur in the undeveloped portions of the islands, partly due to lack of perching structure, but they might be able to establish reproducing populations if introduced to urbanized parts of the islands.

#### Family Polychrotidae

##### Anolis carolinensis

Taxon classificationAnimaliaSquamataPolychrotidae

Voigt, 1832

CB5BA132-21AB-579A-8CD5-B04E7AFDAACB

###### Notes.

*Anoliscarolinensis* is an abundant fixture of urban backyards and woodlands throughout the southeastern United States. There are no museum records from NPI and only two from Mustang Island but there are iNaturalist records from all urbanized areas of the South Texas barrier islands. Across its range, *A.carolinensis* is rarely observed in undeveloped areas and is probably absent from undeveloped portions of the islands due to lack of perching structures. I found 84 museum records from the seven counties.

##### Anolis sagrei

Taxon classificationAnimaliaSquamataPolychrotidae

Duméril & Bibron, 1837

F27C859E-D7DF-5D0F-8881-E6644FDC215A

###### Notes.

*Anolissagrei* is another mostly urban species, native to Cuba and The Bahamas. It was first collected in the United States in Florida in 1935. The first records from Texas were specimens collected in Cameron County in 1986. I found nine museum specimens from the seven counties and 637 iNaturalist observations from the islands since 2009. One hundred nine of those came from NPI, all from the urbanized northern tip of the island.

#### Family Scincidae

##### Plestiodon obsoletus

Taxon classificationAnimaliaSquamataScincidae

Baird & Girard, 1852

7D5AD91B-877A-5061-933D-C0ECA8F9B1E6

**Figure 6.**
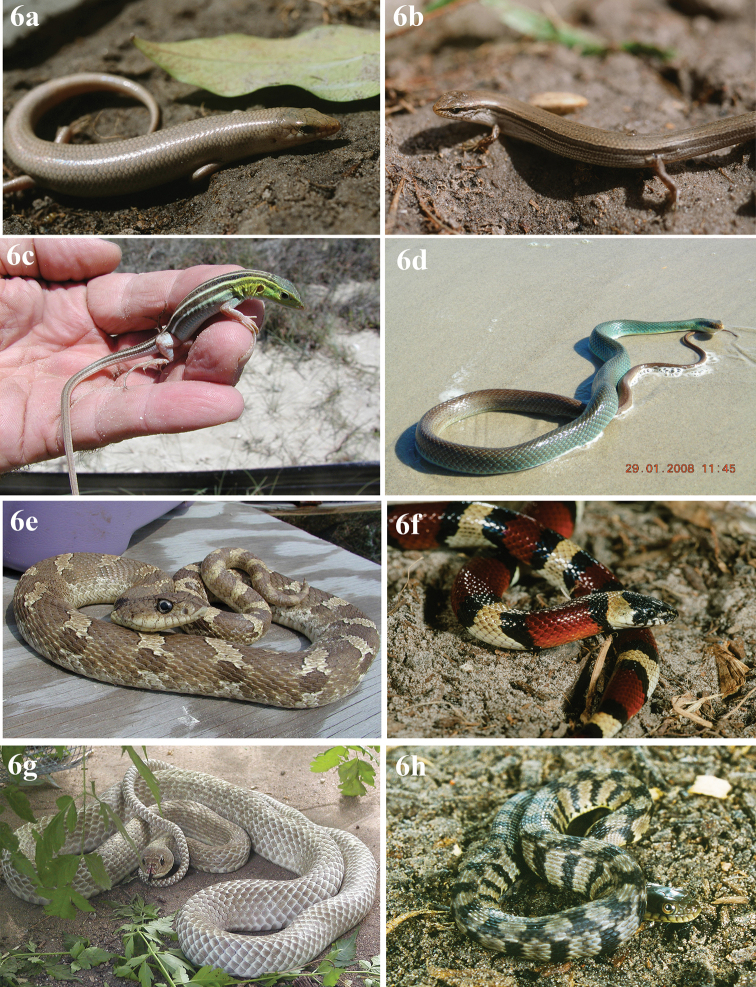
Eight reptiles that occur on North Padre Island **a***Plestiodonobsoletus* (great plains skink **b***Scincellalateralis* (little brown skink) **c***Aspidoscelissexlineatus* (six-lined racerunner) **d***Coluberconstrictor* (North American racer) **e***Heterodonplatirhinos* (eastern hognose snake) **f***Lampropeltistriangulumannulata* (Tamaulipan milksnake) **g***Masticophisflagellum* (coachwhip) **h***Nerodiarhombifer* (diamondback watersnake).

Great Plains skink Fig. 6a

###### Notes.

Our team captured *P.obsoletus* ten times at five different localities from near the northern boundary of PINS to 56 km down the island, in wetlands, xeric grasslands, and dunes. We collected three specimens. There were seven specimens from NPI in museums and 47 specimens from the seven counties but none from South Padre or Mustang Islands. There is one iNaturalist record for Mustang Island based on a photo taken in 1985 (https://www.inaturalist.org/observations/2578244).

##### Scincella lateralis

Taxon classificationAnimaliaSquamataScincidae

Say, in James, 1822

AF886E06-A6DE-5E77-8285-A42C721F0B60

Little brown skink Fig. 6b

###### Notes.

I located four museum specimens and 31 iNaturalist observations of *S.lateralis* from NPI and two museum specimens and only one iNaturalist record from Mustang Island. There are no records from SPI. I found 82 museum specimens from the seven counties. Our team captured *S.lateralis* at seven localities from the northern boundary of PINS to 56 km down the island. All except one of the observations were in moist grasslands/wetlands. The exception was an individual that was trapped at study site 11, which was in sparsely vegetated foredunes.

In earlier versions of their online list, NPS had listed *Plestiodonseptentrionalis* (prairie skink) as a species that occurs on PINS mostly based on a specimen in the NPS vertebrate collection (formerly PAIS 2025; now TCWC 93804) that had been identified as *P.septentrionalis* but has since been reidentified as *S.lateralis.* There is no evidence that the *P.septentrionalis* occurs on the barrier islands but no reason to think that it might not.

#### Family Teiidae

##### Aspidoscelis gularis

Taxon classificationAnimaliaSquamataTeiidae

Baird & Girard, 1852

CDE53024-6DCD-517E-A34D-BE3A92D39E49

###### Notes.

*Aspidoscelisgularis* was classified as “uncommon” by Rabalais (1975). It was not mentioned by Baker and Rabalais (1978). Our team did not observe *A.gularis* during the 2002–2003 surveys. I found two specimens (AMNH 8157, 8158), collected in 1916, and one iNaturalist record (observed in 2017) from South Padre Island. I found three specimens labelled *A.gularis* from NPI (UMMZ 54001 and 54004 and ASNHC 235), but upon examination of photos and consultation with the curators, I determined that all three were *A.sexlineata*. There is one specimen from Mustang Island (TNHC 50473). The Mustang Island specimen is too faded to identify to species, and there is insufficient evidence to dispute the locality, but in my experience, unique localities represented by single specimens are often misidentifications or mislocations. The species is common in the inland portion of the seven counties, where I located 274 museum specimens. In Duran (2004) I reported that I found a museum specimen (AMNH R-168649) 4 km south of the Port Mansfield on South Padre Island, but I later determined that specimen probably came from the mainland side of the Laguna Madre.

##### Aspidoscelis sexlineata

Taxon classificationAnimaliaSquamataTeiidae

Linnaeus, 1766

3B85F1CB-155B-58B1-8180-990C98ED68FD

Six-lined racerunner Fig. 6c

###### Notes.

*Aspidoscelissexlineata* was captured and photographed 46 times. Team members recorded another 16 observations during visual encounter surveys and made many more casual observations. I located 24 specimens in museum collections from NPI, eighteen from Mustang Island, five from South Padre Island, and 145 specimens from the mainland. The species was observed in the back-beach, foredunes, grasslands, emergent wetlands, and dunes.

Trauth (1992) described a subspecies of the six-lined racerunner, the yellow-headed racerunner (*A.s.stephansae*; originally *A.s.stephansi*, emended by Trauth 1995), from Kenedy, Willacy Brooks, and Jim Hogg counties. The localities for some specimens Trauth (1992) examined to describe *A.s.stephansae* were within a sandy ecological zone just across the Laguna Madre from NPI. Trauth (1992) described *A.s.stephansae* as, on average, smaller (< 70 mm) than *A.s.sexlineata* (eastern six-lined racerunner), with a distinctive yellow coloration on the face. Some of the specimens that we captured had bright yellow faces extending from the snout to the nape (Fig. 6c). The snout to vent length was < 70 mm for all specimens during the 2002–2003 surveys. Another characteristic by which Trauth and McAllister (1996) distinguished the subspecies was the relative position of horizontal stripes: In contrast to other *A.sexlineata*, the yellow-headed race purportedly has no vertebral stipe, paravertebral stripes converge just posterior to the rump and extend onto the anterior one fourth of the tail, dorsolateral stripes extend onto the tail, and lateral stripes blend into the bright ventrolateral surface of the tail. Based on limited analysis, I could not determine if the NPI specimens could be assigned to *A.s.stephensae.*

iNaturalist recognizes the taxon, but no observations of *A.s.stephensae* have been entered, while 12 specimens have been identified as *A.sexlineata* within the taxon’s purported range as defined by Trauth (1992). Because identification of the subspecies is based on external morphology and pattern, and apparently no one can identify it based on those characteristics, further analysis is needed to determine if this is a valid taxon.

#### Suborder Serpentes, Family Colubridae

##### Arizona elegans

Taxon classificationAnimaliaSquamataColubridae

Dixon, 1960

E59D5F23-9BE7-5367-AF5C-ABFC5DE1E7F8

###### Notes.

I found eight museum specimens of Arizonaelegans from NPI, six from Mustang Island, and one from SPI (as of 08 October 2020). Another iNaturalist observation from SPI was entered in April 2021 and was deposited into the TNHC collection. I found 36 museum specimens from the seven counties. Since the species is nocturnal and often fossorial, historical records are not good indicators of its relative abundance. During the 2002–2003 surveys, we observed one *A.elegans*. Between 2013 and 2020, seven iNaturalist observations were entered for Mustang Island and six were entered for NPI. Rabalais (1975) referred to *A.elegans* on NPI as “fairly common.” Baker and Rabalais (1978) referred to *A.elegans* as “common” on both NPI and Mustang Island. Glossy snakes in the study area are usually assigned to the subspecies *A.e.arenicolus* (Texas glossy snake).

##### Coluber constrictor

Taxon classificationAnimaliaSquamataColubridae

Linnaeus, 1758

2304ACB6-7734-5223-B8CD-291F4D595576

North American racer Fig. 6d

###### Notes.

I collected one road-killed *C.constrictor* (TCWC 93867) on Park Road 22, north of PINS where it passed through a flooded grassland/wetland. A team member observed one on the beach ~ 3 km south of the southern end of Park Road 22. Figure 6d is a PINS file photo of a *C.constrictor* on the beach, on wet sand near the surf. *Coluberconstrictor* is a habitat generalist, but I did not find any mentions of *C.constrictor* on beaches in the literature. I found three iNaturalist records for *C.constrictor* on beaches in California, Florida, and Virginia. There are two museum specimens and eleven iNaturalist records from NPI but no records of any kind from Mustang or South Padre islands. There is an iNaturalist observation of a bird in flight carrying a *C.constrictor*; the photograph was taken on Mustang Island, but it is impossible to know where the bird caught the snake. There were 83 specimens from the mainland portion of the seven counties. Both Dixon (2013) and Werler and Dixon (2000) show the range of the subspecies, *C.c.oaxaca* (Mexican racer), extending from Mexico northeast along the coast to Aransas County, Texas. The racer I collected (TCWC 93867) was intermediate in key morphometrics between *C.c.oaxaca* and the *C.c.flaviventris* (yellow-bellied racer). Museum specimens of both subspecies are catalogued in each of the seven counties adjacent to the South Texas barrier islands. Werler and Dixon (2000) describe *C.c.flaviventris* as usually having seven supralabial scales and *C.c.oaxaca* as having eight, and both subspecies will usually have 17 or fewer dorsal scale rows at midbody. The specimen collected had a dark green dorsum and yellow ventrum, most like *C.c.flaviventris*, but it had six supralabials on one side and eight on the other and 15 dorsal scale rows, so it could not be assigned to subspecies based on those features. Burbrink et al. (2007) performed genetic analysis which appeared to indicate that *C.constrictor* may be composed of six independently evolving lineages not concordant with most recognized subspecies. No samples within the range of *C.c.oaxaca* were included in that analysis.

##### Drymarchon melanurus

Taxon classificationAnimaliaSquamataColubridae

Cope, 1860

DF346763-E36D-5C88-B342-0E034C24D878

###### Notes.

I found one museum record for the islands (TAMUK 5526), a road-killed snake collected on Park Road 22, just north of the PINS entrance station. That specimen is among numerous specimens from the TAMUK collection (later moved to AMNH) that have been lost. I spoke with the collector and former curators and confirmed that the record is legitimate (Donna Shaver, Allan Chaney, pers. comm.). There were 64 museum records from the seven counties: only three of those were from the counties adjacent to Mustang Island. The Nature Conservancy ecologist, Lee Elliott, captured a *D.melanurus* in the surf near Bob Hall pier on the northern end of NPI and released it in the dunes (Lee Elliott, pers. comm.). Specimens from that part of the range are mostly identified as the subspecies *D.m.erebennus* (Texas indigo snake), which is a large, highly mobile, and conspicuous species. The absence of anecdotal reports and road-kills may indicate that the snake is an occasional visitor to the island. It is a powerful swimmer that probably crosses the Laguna Madre occasionally and might populate the island if conditions there became preferable, but historical and recent evidence does not indicate that it is or has been a permanent resident.

##### Heterodon platirhinos

Taxon classificationAnimaliaSquamataColubridae

Latreille, 1801

0541A98A-A7E9-5F53-B766-BC77BA681726

Eastern hognose snake Fig. 6e

###### Notes.

Our team collected two road-killed specimens (TCWC 93872, TCWC 93873) and captured and examined three others during the 2002–2003 surveys. The road-killed specimens were surrounded by a grassland/wetland matrix. The trapped specimens were all from one location which was in a transition zone between sparsely vegetated foredunes and a more densely vegetated grassland on deep but stable sand. Photos of three of those specimens are the only iNaturalist records for the species from the South Texas barrier islands. There are eight museum specimens from NPI prior to the 2002–2003 surveys but no records of any kind from the South Texas barrier islands after that. Baker and Rabalais (1978) said that *H.platirhinos* on NPI was “one of the more common snakes on Mustang Island, particularly around the town of Port Aransas, which abounds with toads.” The historical record does not support the Baker and Rabalais (1978) assessment, as there are only three museum specimens and no other type of records from Mustang Island. The last of the Mustang Island specimens was collected in 1991. Whether the apparent decline of *H.platirhinos* on Mustang Island is related to the previously discussed extirpation of *Bufospeciosus* (a primary prey item), should be investigated further. I found 15 museum specimens from the mainland portion of the seven counties.

##### Lampropeltis getula

Taxon classificationAnimaliaSquamataColubridae

Linnaeus, 1766

5A0CDB06-4FC3-5A14-9493-C6BBFF5D4CE0

###### Notes.

There are no records for *Lampropeltisgetula* from NPI or SPI. One specimen from NPI (UMMZ 224256) had been labelled “*L.getulus*,” but in consultation with the curator of that collection, I determined that specimen was a mislabeled *L.triangulum*. Within the study area, two species or subspecies have been recognized: *L.g.holbrooki* (Stejneger 1903) and *L.g.splendida* (Baird & Girard 1853). Baker and Rabalais (1978) referred to *L.g.splendida* as “common” on Mustang Island and “possible” for NPI. Pyron and Burbrink (2009) proposed changes to the taxonomy and distribution boundaries of the *Lampropeltisgetula* complex, which would elevate both subspecies to full species status. Widely followed taxonomic sources such as Crother et al. (2017), The Reptile Database (2021), and iNaturalist (2021) have adopted that taxonomy. Following that arrangement has created some confusion about the correct identification and taxonomy of kingsnakes on Mustang Island and the seven counties. Werler and Dixon (2000) noted that the seven counties adjacent to the South Texas barrier islands and much of central Texas lie within a broad area of intergradation between the subspecies, *L.g.splendida* and *L.g.holbrooki*, which led observers and collectors to identify specimens of both species or subspecies in the study area: Thirteen museum specimens were labelled *L.getula* ssp., 26 were labelled *L.holbrooki*, and twelve were labelled *L.splendida*. For this report I retained the subspecies arrangement of Blaney (1977) and used the verbatim museum labels for Table 2. There are 21 iNaturalist observations from Mustang Island identified as *L.holbrooki* and three identified as *L.splendida*. On Mustang Island, morphological characteristics of kingsnakes are sometimes more like L. (g.) holbrooki and sometimes more like L. (g.) *splendida*, but it seems unlikely that there are two independently evolving kingsnake species on the island. Variation in coloration and pattern displayed by kingsnakes on Mustang Island should probably be regarded as phenotypic variations among genetically similar individuals, i.e., morphotypes, not different species.

##### Lampropeltis triangulum

Taxon classificationAnimaliaSquamataColubridae

Kennicott, 1861

1C0DE85A-B79E-55AB-A5C1-6B4921D03A31

Milksnake Fig. 6f

###### Notes.

Our team observed four *L.triangulum* and took two as specimens (TCWC 93878, 93879) during the 2002–2003 surveys. Those observations were all within the grassland wetland matrix. Following the taxonomic arrangement prevalent at the time (Williams 1978), I labelled those specimens *L.t.annulata*. Ruane et al. (2014) proposed reducing the 14 previously recognized subspecies of *L.triangulum* to six species and redefined the distribution of those species. Widely followed taxonomic sources like Crother et al. (2017), iNaturalist (2021), and The Reptile Database (2021) adopted the Ruane et al. (2014) proposals. According to that arrangement, the species that occupied the seven counties would become *L.annulata*. Chambers and Hillis (2020) questioned the validity of the Ruane et al. (2014) analysis, stating that “over-reliance on the program Bayesian Phylogenetics and Phylogeography (BPP), without adequate consideration of its assumptions and of sampling limitations, resulted in over-splitting of species in this study.” Chambers and Hillis (2020) did not propose a new taxonomic arrangement but demonstrated that the BPP program can be used to support “virtually *any* geographic partition of samples in this potential continental cline as species.” Of the 14 specimens from NPI in museum databases, eight were originally labelled *L.t.annulata*, five were labelled *L.triangulum* ssp. and one was labelled *L.t.gentilis*. One museum specimen from Mustang Island was labelled *L.t.annulata* and three were labelled *L.triangulum* ssp. In Table 2, I retain the verbatim museum labels. On iNaturalist, which is following the Ruane et al. (2014) arrangement, two photos from Mustang Island have been identified as *L.gentilis*. The nearest mainland iNaturalist observation of *L.gentilis* is near Somerville, Texas, 323 km from the Mustang Island observations.

##### Masticophis flagellum

Taxon classificationAnimaliaSquamataColubridae

Say, in James, 1822

6E37FA8D-FD4A-579D-9910-6119E68BC300

Coachwhip Fig. 6g

###### Notes.

*Masticophisflagellum* was the most observed and most captured snake species during the 2002–2003 surveys. It was trapped 21 times at seven study sites, which spanned 94 km and four ecological zones: grasslands, emergent wetlands, sparsely vegetated foredunes, and dune/swell complexes. from Study Site 1 near Bird Island Basin to Study Site 17, near the Mansfield Channel. I took one specimen (TCWC 93880). There were 25 museum specimens from NPI, thirteen from Mustang Island, and 148 from the seven counties. There are 61 iNaturalist observations from NPI, 25 from Mustang Island, and five from SPI.

##### Nerodia rhombifer

Taxon classificationAnimaliaSquamataColubridae

Hallowell, 1852

2BAD0312-A726-5589-BE39-65FD3110C36A

Diamondback watersnake Fig. 6h

###### Notes.

*Nerodiarhombifer* was observed four times and captured once near the man-made pond along the road to Bird Island Basin. There were two museum specimens from NPI and none from Mustang Island. There are four iNaturalist record for NPI, and none from Mustang Island. I found 163 specimens from the mainland portion of the seven counties. Mostly a fish eater and dependent on permanent fresh water, the species is probably only found on the northern end of NPI in and around the several manmade ponds.

##### Nerodia clarkii

Taxon classificationAnimaliaSquamataColubridae

Baird & Girard, 1853

1827CEC1-DB3B-5E9E-9EB2-03EEC975E07D

###### Notes.

*Nerodiaclarkii* is regularly observed along the shorelines of Nueces, Corpus Christi, and Oso bays, and Mustang Island, but an iNaturalist observation of a road-killed specimen, near the northern end of NPI, just south of the Nueces County line, is the only verifiable occurrence of the species on NPI or in Kleberg County (https://www.inaturalist.org/observations/8105046). There are 33 iNaturalist observations and 18 museum records from Mustang Island. Forty-three more museum specimens have been taken around Corpus Christi Bay, in the counties of Nueces, San Patricio, and Aransas. The southern edge of the range of the species is an elastic boundary where the brackish water of Corpus Christi Bay meets the hypersaline water of the Laguna Madre. *Nerodiaclarkii* could enter the upper Laguna Madre when higher precipitation lowers salinity, then retreat into Corpus Christi Bay when salinity rises, an example of “behavioral osmoregulation” (Dunson and Mazzotti, 1989).

##### Cemophora lineri

Taxon classificationAnimaliaSquamataColubridae

Weinell & Austin 2017

012C814F-0F72-50E3-BE1B-C4E7B335FF62

**Figure 7.**
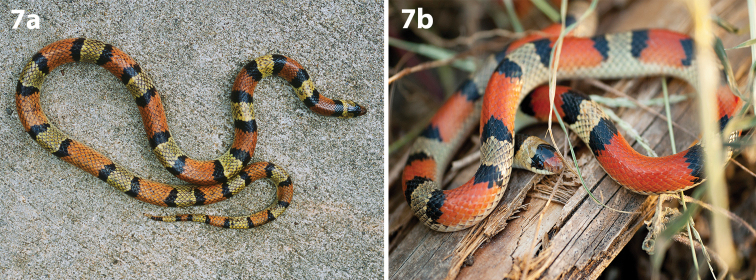
Comparison of *Cemophoralineri* (Texas scarletsnake) specimen from North Padre Island with *C.lineri* specimen from San Patricio County **a***Cemophoralineri* (Texas scarletsnake) from North Padre Island **b***Cemophoralineri* from San Patricio County. Note that the colors of the NPI specimen are duller than those of the scarletsnake from the mainland.

Texas scarletsnake Fig. 7a, b

###### Notes.

There is only one record for *Cemophoralineri* from NPI or the South Texas barrier islands, an individual I captured in a grassland near the mid-point between the end of Park Road 22 and the Mansfield Channel in 2002 (photo voucher, TNHC 86866; Fig. 7a). Seemingly rare throughout the range, like other fossorial snakes, *C.lineri* may be more common than the sparse historical record implies; hundreds of square kilometers of suitable habitat within its range are privately-owned and largely inaccessible. It is listed as “threatened” by the state of Texas. Because of its rarity and the unique nature and atypical morphology of this lone barrier island specimen, a more detailed discussion follows.

Aufenburg (1948) collected the first specimens of what were probably *C.lineri* on U.S. Naval Air Station, Corpus Christi, on the mainland, near the northern end of NPI. At that time the nearest known occurrence of *Cemophora* sp. was > 1000 km from Auffenberg’s observations, so it is difficult to fault Aufenburg for stating: “There is no doubt that these snakes were accidentally brought in from Pensacola, Florida, from which station we received much air cargo.” It is not impossible that Auffenberg (1948) was right about the source of those snakes and the specimens were lost before they could be analyzed (Williams et al. 1966, citing personal communication with Auffenberg). Referring to that possibility, Brown (1950) called it “a doubtful species,” but most researchers later concluded that those specimens were probably from southern Texas (Williams et al. 1966; Williams and Wilson 1967).

Williams et al. (1966) found that scutellation and other morphological features of the only two *Cemophora* specimens from southern Texas (AMNH 75307 [holotype] and BCB 10993 [paratype]) were distinctly different from *Cemophora* specimens known from the southeastern United States and described a new subspecies, *C.coccinealineri* (Texas scarletsnake). During a more extensive review of the genus, Williams and Wilson (1967) confirmed that morphometrics of C. (c.) lineri were different from the two previously recognized subspecies, *C.c.coccinea* (Florida scarletsnake) and *C.c.copei* (northern scarletsnake).

Williams et al. (1966) reported that one of the more distinct differences between C. (c.) lineri and the southeastern *Cemophora* was the significantly higher number of ventral scale rows (VSR) for C. (c.) lineri: They found that the VSR count of 59 specimens of *C.c.coccinea* was 158–185 (x̄ = 174.0) and VSR count of 180 *C.c.copei* specimens was 150–180, (x̄ = 165.3). For the two C. (c.) lineri specimens available for the original description, VSR were 188 and 195, (x̄ = 191.5). The comparisons led Williams and Wilson (1967) to hypothesize that C. (c.) lineri was more closely related to the more geographically distant *C.c.coccinea* than to the nearer *C.c.copei*, which they attributed to climactic conditions that led to a splitting of *C.coccinea* during the Pleistocene. Weinell and Austin (2017) proposed elevating the subspecies to *C.lineri* based on their genetic analysis; that analysis indicated that *C.lineri* diverged from the *C.coccinea* in the Pliocene or early Pleistocene and that *C.lineri* is monophyletic, while *C.c.coccinea* and *C.c.copei* are paraphyletic. Crother et al. (2017) and other taxonomic sources adopted that taxonomy.

In addition to their genetic work, Weinell and Austin (2017) performed a phenotypic analysis of five southern Texas specimens (including the NPI specimen: TNHC 86866) and the two specimens used by Williams et al. (1966) in the original description. In rough concurrence with Williams et al. (1966), they found that *C.lineri* differed most distinctly from *C.coccinea* in the number of VSR (178–195; x̄ = 186.1). The results of that analysis provided evidence that the forms are morphologically dissimilar as well as genetically distant from the southeastern *Cemophora*. In reporting the VSR count, Weinell and Austin (2017) included the VSR count for the NPI specimen (178). Excluding the NPI specimen from that analysis would leave its VSR count outside the range of variation of other known specimens of *C.lineri* but within the range of variation and near the mean for *C.c.coccinea*. There are no published data on *C.lineri* morphometrics which includes more specimens, but in unpublished notes, The Nature Conservancy zoologist, John Karges, analyzed eleven morphometric features of the ten *C.lineri* specimens residing in natural history collections in the late 1970s (John Karges, pers. comm.): the VSR count of the NPI specimen is still well outside of the range of variation in VSR in that larger dataset (183–197, x̄ = 188.5).

As it is with other reptiles and amphibians on NPI, the colors of the *C.lineri* I photographed on NPI are duller than those of other *C.lineri* I have observed in the study area on the mainland. Figure 7a is the snake I captured on NPI and Figure 7b is a *C.lineri* I photographed in San Patricio County.

##### Pantherophis emoryi

Taxon classificationAnimaliaSquamataColubridae

Baird & Girard, 1853

19B78C05-8210-55CE-BCD3-8C8440D95D46

**Figure 8.**
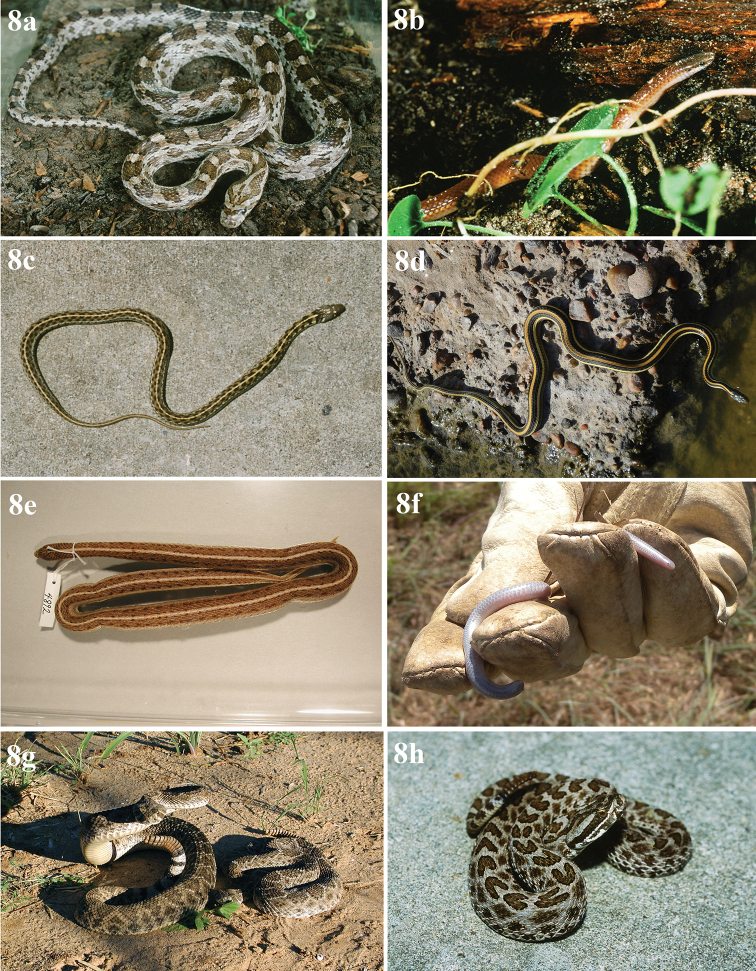
Eight reptiles that occur on North Padre Island **a***Pantherophisemoryi* (Great Plains ratsnake) **b***Tantillagracilis* (flathead snake) **c***Thamnophismarcianus* (checkered garter snake) **d***Thamnophisproximus* (western ribbon snake) **e***Tropidoclonionlineatum* (lined snake) from South Bird Island, just offshore of NPI**f***Renadulcis* (Texas threadsnake; PINS file photo) **g***Crotalusatrox* (western diamond-backed rattlesnake) **h***Sistrurustergeminus* (western massasauga).

Great Plains ratsnake Fig. 8a

###### Notes.

During the 2002–2003 surveys, our team collected two road-killed *P.emoryi* of the four NPI specimens in museum databases (TNHC 85143, TCWC 93868). The road-kills were surrounded by inundated emergent wetlands within a grassland/wetland matrix. Our team captured and photographed three more specimens at two study sites; both sites were on the edge of an inundated emergent wetland surrounded by an extensive grassland/wetland matrix. Between 2015 and 2020, nineteen iNaturalist observations from NPI and 19 from Mustang Island were entered. Rabalais (1975) characterized the species as “possible.” It was not mentioned by Baker and Rabalais (1978). I found 147 museum specimens from mainland portion of the seven counties.

##### Pituophis catenifer

Taxon classificationAnimaliaSquamataColubridae

Schlegel, 1837

551657BB-885D-5F0C-93B3-00784574E791

###### Notes.

I found no museum specimens of *P.catenifer* from the barrier islands, but an iNaturalist record of a 2006 observation (https://www.inaturalist.org/observations/2580986) was entered in 2016. Subsequently, three iNaturalist observations from the northernmost, urbanized part of the island, were entered for snakes observed in 2015, 2019, and 2020. Rabalais (1975) referred to *P.catenifer* as “uncommon.” Given the sparse historical record, *P.catenifer*, a large-bodied snake that is probably capable of swimming across the Laguna Madre, may occur only as a vagrant on the islands, but further study might reveal that it occurs as a reproducing population. I located 74 museum specimens from the inland portion of the seven counties. In southern Texas, specimens are generally identified as the subspecies, *P.c.sayi* (bullsnake).

##### Storeria dekayi

Taxon classificationAnimaliaSquamataColubridae

Holbrook, 1839

3B40C830-A821-516B-8A9F-EA6C02F57A64

###### Notes.

There is one 1982 museum specimen of *S.dekayi* from NPI, which is the only specimen from the South Texas barrier islands. Our team did not detect the species during the 2002–2003 surveys. There are now 13 iNaturalist records from NPI, one from Mustang Island, and one from SPI. I found 188 museum specimens from the inland portion of the seven counties.

##### Tantilla gracilis

Taxon classificationAnimaliaSquamataColubridae

Baird & Girard, 1853

B6EFD439-B006-52A3-A7CF-AF7291EB94D4

Flathead snake Fig. 8b

###### Notes.

*Tantillagracilis* is largely fossorial, thus rarely observed, so they are probably more common on NPI than the three museum specimens and nine iNaturalist observations suggest. During the 2002–2003 surveys, our team captured three *T.gracilis* (TCWC 93900) at three study sites, all within the grassland/wetland matrix. There are no records for Mustang or South Padre islands. I found 52 *T.gracilis* museum specimens from the inland portion of the seven counties.

##### Thamnophis marcianus

Taxon classificationAnimaliaSquamataColubridae

Baird & Girard, 1853

94B0B674-1641-59F1-9F90-0FA81AE13C52

Checkered gartersnake Fig. 8c

###### Notes.

*Thamnophismarcianus* is common and conspicuous across most of its range. Our team observed this species three times. One road-killed specimen (TCWC 93901) was collected. I found eight museum specimens from NPI, fourteen from Mustang Island, one from Harbor Island (near Port Aransas), and 327 records from the inland portion of the seven counties. There are ten iNaturalist observations for Mustang Island and 33 from NPI. All observations are closely associated with wetlands and ponds within the grassland/wetland matrix. Its distribution is probably limited by the availability of freshwater to the northern 27 km of the island.

##### Thamnophis proximus

Taxon classificationAnimaliaSquamataColubridae

Rossman, 1963

8E0A9B7C-7D99-59C5-97F0-CD400C192CED

Western ribbonsnake Fig. 8d

###### Notes.

During the 2002–2003 survey our team captured *Thamnophisproximus* four times and collected six road-killed specimens (TCWC 93902–93908). All observations were closely associated with emergent wetlands within the grassland/wetland matrix. Baker and Rabalais (1978) reported that the species was more common on Mustang Island than on NPI, but museum and iNaturalist observations indicate that the species is and has been more common on NPI. I found 17 museum records and 413 iNaturalist observations from NPI. There was one museum specimen and 41 iNaturalist observations from Mustang Island. The distribution of this semi-aquatic fish-eating species on NPI is probably limited to the northern 27 km of the island by the availability of freshwater. Specimens that occur in the area are usually identified as the subspecies, *T.p.orarius* (Gulf Coast ribbonsnake).

##### Tropidoclonion lineatum

Taxon classificationAnimaliaSquamataColubridae

Hallowell, 1856

152704DA-C22F-59B5-93C2-858A9C11CF4C

Lined snake Fig. 8e

###### Notes.

A single museum specimen of *Tropidoclonionlineatum* (AMNH 171739) was collected in 1980 on South Bird Island, a 10.9 ha island in the Laguna Madre, ~ 75 m from NPI and ~ 2.0 km northwest of the western end of Bird Island Basin Road. There are no other records of *T.lineatum* from the South Texas barrier islands or from the seven adjacent counties (Werler and Dixon 2000; Dixon 2013). This locality is ~ 138 km from the nearest locality to the northeast in Calhoun County and ~ 156 km from the nearest locality to the northwest in Duval County. I could not locate the collectors (Richard R. Schmidt and C. Byrd) for comment. The species is spottily distributed from the north-central United States to south-central Texas.

#### Family Leptotyphlopidae

##### Rena dulcis

Taxon classificationAnimaliaSquamataLeptotyphlopidae

Baird & Girard, 1853

BF6541DB-6830-5DE2-A4CF-FBCB2F202BE5

Texas threadsnake Fig. 8f

###### Notes.

*Renadulcis* is known from the South Texas barrier islands from one photo taken by PINS staff in 2010. That snake was uncovered ~ 46 cm underground while digging a hole for a fence post. There is an iNaturalist record from the north end of Mustang Island, but the photo submitted with that record is not detailed enough to determine with certainty if the snake is *R.dulcis* or the non-native *Indotyphlopsbraminus* (Brahminy threadsnake; https://www.inaturalist.org/observations/1270983). There are 101 museum records from the seven counties. *Renadulcis* is fossorial and not easily observed, so it is probably more common on NPI (and possibly Mustang Island) than the sparse historical record implies.

#### Family Viperidae

##### Crotalus atrox

Taxon classificationAnimaliaSquamataViperidae

Baird & Girard, 1853

1440E3A6-1C94-53FF-8BB5-FC02B062BEA1

Western diamondback rattlesnake Fig. 8g

###### Notes.

Baker and Rabalais (1978) reported that the western diamondback rattlesnake was “very common” on NPI and Mustang islands from the foredunes through the vegetated barrier flats. We received a few anecdotal reports and one photo during the 2002–2003 surveys, but our team did not observe *C.atrox*. I found three museum specimens and seven iNaturalist records from the northern end of NPI, seven museum specimens and 28 iNaturalist records from Mustang Island, one iNaturalist records from SPI, and 358 museum specimens from the seven counties. While it is clearly not uncommon, the historical record does not support the Baker and Rabalais (1978) contention that *C.atrox* is “very common.” I have avoided the use of subjective classifications of abundance, but historical records for *C.atrox* are sparse compared to species such as *Masticophisflagellum* or *Holbrookiapropinqua*. Observing this large venomous reptile may be frightening and memorable, and observations may be reported and repeated out of proportion to its abundance.

##### Sistrurus tergeminus

Taxon classificationAnimaliaSquamataViperidae

Say, 1823

0B2C9487-26D2-5D6B-90B8-FB87D2D5C261

Western Massasauga Fig. 8h

###### Notes.

During the 2002–2003 surveys, out team captured five *S.tergeminus*. I located 19 museum specimens and three iNaturalist records from NPI but no records from Mustang or South Padre islands. There are ten museum specimens and one iNaturalist observation from the mainland portion of the seven counties. Baker and Rabalais (1978) reported that *S.catenatus* (*S.tergeminus*) was common on NPI but unknown from Mustang Island. At the time of the 2002–2003 surveys, two subspecies, *S.t.tergeminus* and *S.t.edwardsi* were recognized, but the snakes observed were morphologically intermediate between those subspecies, per the diagnostic characteristics described by Werler and Dixon (2000) and could not be assigned to subspecies. Kubatko et al. (2011) and Ryberg et al. (2015) report that the genetic distance between *S.t.tergeminus* and *S.t.edwardsi* is not significant. Currently most authoritative sources do not follow the subspecies arrangement.

## Summary and discussion

Given the dynamic nature of barrier island geomorphology and the resulting equally dynamic arrangement of ecological zones, it is not surprising to find evidence of fluctuations in the occurrence and abundance of herpetofauna over the time-period for which we have records. Some species said to be abundant in the historical record were found to be rare or absent during all or part of this study period (2002–2020), and some species found to be abundant during the study period were absent or sparsely represented in the historical record. In some cases, failure to observe a species for decades has led me to presume they have been extirpated. The variability between time periods is particularly evident for Bufonids (true toads). Moore (1976) reported that the Texas toad (*Bufospeciosus*) was the most abundant anuran on Mustang Island during his 1971 study, but with no verifiable records since 1970, *B.speciosus* appears to have been extirpated from Mustang Island (there is no evidence it ever occurred on NPI or SPI). The gulf coast toad (*B.nebulifer*), which is quite conspicuous when present and abundant on the mainland, was not detected during the 2002–2003 surveys. There were no records for North Padre Island between 1891 and 2007 and not another until 2017 when choruses could be heard within the city limits of Corpus Christi on northernmost Padre Island. Woodhouse’s toad (*B.woodhousii*) was not known from the islands until our team collected and audio-recorded it numerous times during the 2002–2003 surveys; it was not reported again until 2018. In a region known for extended droughts, *B.nebulifer* and *B.woodhousii* may fail to reproduce in some years and may experience periodic population declines that make them nearly undetectable (Perchman and Wilbur 1994; Green 2003; Brown et al. 2012). The natural history of Bufonids on the South Texas barrier islands, particularly examining how their prey and predator communities and their preferred habitats have been altered, warrants further study. Determining the mechanisms of the fluctuations would help inform conservation actions for the species. The eastern hognose snake (*Heterodonplatirhinos*), which preys on Bufonids, also appears to experience noticeably fluctuating population densities: the species was consistently observed and collected from the 1960s through the 1990s, and our team recorded five individuals during the 2002–2003 surveys, but no *H.platirhinos* have been reported from the islands since that time. The last *H.platirhinos* specimen collected from Mustang Island was in 1991, a few years after *B.speciosus* appears to have been extirpated. While the evidence for interacting population fluctuations between Bufonids and *H.platirhinos* on the islands is circumstantial, further study is needed. The most high-profile extirpation is that of the Texas horned lizard (*Phrynosomacornutum*), which is listed as an endangered species by the state of Texas and is the Texas state lizard. Baker and Rabalais (1978) referred to *P.cornutum* as the most common lizard on Mustang Island. It is still present on nearby San José Island, on another small island in Corpus Christi Bay, and on the mainland. Natural or human-aided repopulation of the species on Mustang Island might be possible if the threats which led to the extirpation are identified and mitigated.

Non-marine species that are extirpated from islands may later repopulate naturally despite the ecological barrier posed by saltwater. Baker and Rabalais (1978) reported finding a Texas tortoise (*Gopherusberlandieri*) and a common snapping turtle (*Chelydraserpentina*) dead on the beach and speculated that some turtles arrive there after being washed into the surf and carried along by longshore currents. Additional evidence supports that hypothesis: In 2007 and again in 2021, a live alligator, which had been tagged in Louisiana, washed up on an NPI beaches. In 2008, tons of debris, much of it lumber from destroyed houses, washed up on NPI beaches a few days after hurricane Katrina struck Louisiana and Mississippi (while that is the most extreme, debris from Gulf Coast storms commonly washes up on NPI beaches). In an extensive review of the literature, Neill (1958) provides many examples of non-marine amphibians and reptiles living in or adapting to saline environments including a common slider (*Trachemysscripta*) trapped in a brackish canal in the Sabine Wildlife Refuge (Cagle and Chaney 1950) and a speckled kingsnake (*Lampropelisgetulaholbrooki*) in brackish water in Cameron Parish, Louisiana (he cited personal communication with JR Dixon). On Merritt Island, Florida, Neill (1958) observed southern leopard frogs (*Ranasphenocephala*) and green treefrogs (*Hylacinerea*) jumping into water “too salty to drink” He also heard eastern narrow-mouthed toads (*Gastrophrynecarolinensis*) calling from *Salicornia* flats, and he collected *C.serpentina* on a tidal flat. Sissom et al. (1990) reported that one of the three man-made ponds within PINS was “too salty to be classified as freshwater,” and that they did not observe any vertebrates using the pond. However, we trapped *T.scripta* in hoop traps on each of four trap days and observed three introduced *A.mississippiensis* (frequently found in brackish water) in the pond. I found this iNaturalist observation, entered by The Nature Conservancy Zoologist John Karges, of a slender glass lizard (*Ophisaurusattenuatus*) swimming ~ 100 m from shore near Port O’Conner (https://www.inaturalist.org/observations/82288781) and this observation of *Masticophisflagellum* swimming 1.2 km from shore in Mesquite Bay (https://www.inaturalist.org/observations/95038106). The Nature Conservancy biologist, Lee Elliott, observed an indigo snake (*Drymarchonmelanurus*) swimming in the surf near Bob Hall Pier on North Padre Island (Lee Elliott, pers. comm.). That species is large, conspicuous, and highly mobile. It is relatively common on the mainland and may occur on the island accidentally. Similarly, the gopher snake (*Pituophiscatenifer*), another large, conspicuous snake, for which there were anecdotal accounts but no museum records or verifiable observations until 2006, may occur on the island occasionally or accidentally. The unexpected appearance of a diamondback terrapin (*Malaclemysterrapin*) on the seaward side of southernmost Padre Island, might be a natural range expansion, human-aided introduction, or another example of a reptile being carried outside of its native range by Gulf of Mexico currents.

Several species found on NPI differ in relative abundance and/or morphology from their mainland counterparts. Padre Island herpetofauna are generally paler than mainland specimens; in particular, *B.woodhousii*, *M.flagellum*, and the great plains ratsnake (*Pantherophisemoryi*) found on the island exhibit a ground color that is often nearly white, and the reds and yellows of the Texas scarletsnake (*Cemophoralineri*) that we observed were duller than its mainland counterpart. The ventral scale row count of one *C.lineri* specimen was found to be outside of the range of variation for that species but within the range of variation and near the mean for *C.c.coccinea* (this may be a single aberrant individual). The only Ranid found in the mainland counties adjacent to Padre Island is the Rio Grande leopard frog (*Ranaberlandieri*), while both *R.berlandieri* and *R.sphenocephala* are found on NPI. The external morphology of some Ranid specimens found on North Padre Island are intermediate between *R.berlandieri* and *R.sphenocephala*, therefore calls are the most definitive record of occurrence for those species (Suppl. material 4). Prior to a single recent iNaturalist record, the only Gastrophrynid known from the South Texas barrier islands was *G.carolinensis*, while only the western narrow-mouthed toad (*G.olivacea*) was known from the inland counties. Additionally, *G.carolinensis* is found in the Kleberg County portion of NPI, while that species is not found on the adjacent mainland in Kleberg County.

Several non-native species of amphibians and reptiles have established breeding populations in southern Texas. Two non-native lizards, the Mediterranean gecko (*Hemidactylusturcicus*) and the brown anole (*Anolissagrei*) have well-established populations in southern Texas, including many records from the South Texas barrier islands. *Anolissagrei* are known to displace native green anoles (*A.carolinensis*). iNaturalist records have been posted for the Cuban treefrog (*Osteopilusseptentrionalis*) on the southern end of South Padre Island. There are no other records for that species within 480 km of Corpus Christi, Texas, but the species is well-established in Florida and along the Gulf Coast. The invasion of Florida by Cuban treefrogs has severely impacted native ecosystems and has led to localized extirpations of other frogs in urban areas (Johnson 2017). Texas and federal wildlife agencies and organizations should proactively develop recommendations and protocols for dealing with an inevitable invasion by that species. The Brahminy blindsnake (*Indotyphlopsbraminus*) is well-established in Cameron and Hidalgo counties and a single iNaturalist record for South Padre Island was entered in 2015. The American bullfrog (*R.catesbeiana*) is an invasive species in the western United States where it competes with and preys on native species. While Padre Island is nestled within the native range of *R.catesbeiana*, its appearance on the island, documented in 2018 by an audio recording of a single individual, might be considered invasive.

There were a few species that appeared on previous checklists for NPI that our team did not detect. While it is unlikely that any of the unconfirmed species occur in abundance, it is possible that some of the species may yet be found on the island. The Texas coral snake (*Micrurustener*) probably appeared on previous checklists of North Padre Island herpetofauna (Rabalais 1975; NPS 1984) because of misinterpretation of locality information of one specimen collected at the PINS headquarters when it was on the mainland in Corpus Christi. While little typical habitat for *M.tener* is found on NPI, it is possible that *M.tener* may yet be observed on the island. Couch’s spadefoot (*Scaphiopuscouchii*) appeared on a previous checklist because Baker and Rabalais (1978) said that a specimen had been collected on NPI, but I found no specimens or other verifiable evidence of its occurrence there. Likewise, *B.speciosus* was common on Mustang Island until the mid-1970s, so Rabalais (1975) and Baker and Rabalais (1978) understandably thought it might occur on NPI. While there is no evidence that *S.couchii* or *B.speciosus* ever occurred on NPI, they are common on the mainland and may yet be detected on the island. The lesser siren (*Sirenintermedia*) and the spotted newt (*Notophthalmusmeridionalis*) appeared on previous checklists because of their nearby occurrence on the mainland. Those species require poorly drained, generally clayey, soils (Judd 1983; Gelback et al. 1991). Most NPI soils are sandy but some ponds in the northern 27 km of the island are poorly drained because their substrates are covered in layers of decaying vegetation. Those ponds are difficult to access, and our sampling efforts were insufficient to say with certainty that *S.intermedia* and *N.meridionalis* do not occur on the islands. The lack of verifiable records for the common whiptail (*Aspidoscelisgularis*) is a bit of a mystery. It was listed by Rabalais (1975) as uncommon and Allan Chaney told me he thought he had observed it, but I did not observe it and could find no evidence that it occurs there.

The basic methodology of the 2002–2003 study was completed by the US National Park Service in consultation with their partners prior to my being tasked with coordinating the inventory There were some things I wish I could have done differently. In short, the original design called for random selection of study sites, where we would conduct drift-fence/pitfall trapping in the spring and early summer only. It is standard practice to employ a random sampling scheme in hopes that future researchers will be able to mimic the methodology, but this study area is 50,000 ha, it extends for 122 km, resources were limited, and the primary objective was to determine presence/absence. The freedom to directly target specific species in specific areas at specific times, should have been a primary component of the plan. Random selection, stratified by ecological zones and geography, also caused study areas to be unevenly distributed within stratifications, leaving some gaps that were under-sampled. While spring sampling is a standard feature of most amphibian and reptile and monitoring plans in much of the United States, in coastal southern Texas, where more than 30% of rainfall usually occurs in September and October, a more effective plan would have specified that some drift-fence/pitfall trapping would occur in the fall. While we did continue with visual encounter and calling-frog surveys throughout the year, we were only allowed to conduct drift-fence/pitfall trapping in the spring and early summer.

## Supplementary Material

XML Treatment for Bufo nebulifer

XML Treatment for Bufo speciosus

XML Treatment for Bufo woodhousii

XML Treatment for Gastrophryne carolinensis

XML Treatment for Gastrophryne olivacea

XML Treatment for Eleutherodactylus campi

XML Treatment for Eleutherodactylus planirostris

XML Treatment for Osteopilus septentrionalis

XML Treatment for Hyla cinerea

XML Treatment for Hyla squirella

XML Treatment for Pseudacris clarkii

XML Treatment for Rana berlandieri

XML Treatment for Rana catesbeiana

XML Treatment for Rana sphenocephala

XML Treatment for Scaphiopus couchii

XML Treatment for Scaphiopus hurterii

XML Treatment for Chelydra serpentina

XML Treatment for Malaclemys terrapin

XML Treatment for Terrapene carolina

XML Treatment for Terrapene ornata

XML Treatment for Trachemys scripta

XML Treatment for Kinosternon flavescens

XML Treatment for Gopherus berlandieri

XML Treatment for Alligator mississippiensis

XML Treatment for Ophisaurus attenuatus

XML Treatment for Hemidactylus turcicus

XML Treatment for Holbrookia propinqua

XML Treatment for Holbrookia subcaudalis

XML Treatment for Phrynosoma cornutum

XML Treatment for Anolis carolinensis

XML Treatment for Anolis sagrei

XML Treatment for Plestiodon obsoletus

XML Treatment for Scincella lateralis

XML Treatment for Aspidoscelis gularis

XML Treatment for Aspidoscelis sexlineata

XML Treatment for Arizona elegans

XML Treatment for Coluber constrictor

XML Treatment for Drymarchon melanurus

XML Treatment for Heterodon platirhinos

XML Treatment for Lampropeltis getula

XML Treatment for Lampropeltis triangulum

XML Treatment for Masticophis flagellum

XML Treatment for Nerodia rhombifer

XML Treatment for Nerodia clarkii

XML Treatment for Cemophora lineri

XML Treatment for Pantherophis emoryi

XML Treatment for Pituophis catenifer

XML Treatment for Storeria dekayi

XML Treatment for Tantilla gracilis

XML Treatment for Thamnophis marcianus

XML Treatment for Thamnophis proximus

XML Treatment for Tropidoclonion lineatum

XML Treatment for Rena dulcis

XML Treatment for Crotalus atrox

XML Treatment for Sistrurus tergeminus

## Supplementary materials

Supplementary material 1 Museum and iNaturalist records for Aransas, Cameron, Kenedy, Kleberg, Nueces, San Patricio, and Willacy counties, Texas, and iNaturalist records for North and South Padre and Mustang islands, Texas Data type Collections. Explanation note This is an excel file containing attributes of all museum and iNaturalist records for Aransas, Cameron, Kenedy, Kleberg, Nueces, San Patricio and Willacy counties, Texas, including North and South Padre and Mustang islands. https://binary.pensoft.net/file/616302This dataset is made available under the Open Database License (http://opendatacommons.org/licenses/odbl/1.0/). The Open Database License (ODbL) is a license agreement intended to allow users to freely share, modify, and use this Dataset while maintaining this same freedom for others, provided that the original source and author(s) are credited.Mike Duran

Supplementary material 2 *Gastrophrynecarolinensis* calling, 08 July 2003. Data type Sound file. Explanation note Audio of *Gastrophrynecarolinensis* calling on the night of July 8, 2003. https://binary.pensoft.net/file/616303This dataset is made available under the Open Database License (http://opendatacommons.org/licenses/odbl/1.0/). The Open Database License (ODbL) is a license agreement intended to allow users to freely share, modify, and use this Dataset while maintaining this same freedom for others, provided that the original source and author(s) are credited.Mike Duran

Supplementary material 3 *Hylasquirella* calling 08 July 2003 Data type Sound file. Explanation note Audio of *Hylasquirella* calling on the night of July 8, 2003. https://binary.pensoft.net/file/616304This dataset is made available under the Open Database License (http://opendatacommons.org/licenses/odbl/1.0/). The Open Database License (ODbL) is a license agreement intended to allow users to freely share, modify, and use this Dataset while maintaining this same freedom for others, provided that the original source and author(s) are credited.Mike Duran

Supplementary material 4 *Ranasphenocephala* and possibly *R.berlandieri* calling, 10 February 2013 Data type Sound file. Explanation note Audio of Ranasphenocephala and possibly R.berlandieri calling on the night of February 10, 2013. https://binary.pensoft.net/file/616348This dataset is made available under the Open Database License (http://opendatacommons.org/licenses/odbl/1.0/). The Open Database License (ODbL) is a license agreement intended to allow users to freely share, modify, and use this Dataset while maintaining this same freedom for others, provided that the original source and author(s) are credited.Mike Duran
